# Size does matter: Parallel evolution of adaptive thermal tolerance and body size facilitates adaptation to climate change in domestic cattle

**DOI:** 10.1002/ece3.4550

**Published:** 2018-10-05

**Authors:** Muhammed Elayadeth‐Meethal, Aravindakshan Thazhathu Veettil, Shane K. Maloney, Nichola Hawkins, Tom H. Misselbrook, Veerasamy Sejian, M. Jordana Rivero, Michael R. F. Lee

**Affiliations:** ^1^ Kerala Veterinary and Animal Sciences University Wayanad India; ^2^ School of Human Sciences University of Western Australia Crawley Australia; ^3^ Rothamsted Research North Wyke UK; ^4^ Rothamsted Research Harpenden UK; ^5^ ICAR‐National Institute of Animal Nutrition and Physiology Bangalore India; ^6^ Bristol Veterinary School University of Bristol Langford UK

**Keywords:** adaptive tolerance, heat stress, livestock, phenotypic plasticity, phylogeny, size dependence

## Abstract

The adaptive potential of livestock under a warming climate is increasingly relevant in relation to the growing pressure of global food security. Studies on heat tolerance demonstrate the interplay of adaptation and acclimatization in functional traits, for example, a reduction in body size and enhanced tolerance in response to a warming climate. However, current lack of understanding of functional traits and phylogenetic history among phenotypically distinct populations constrains predictions of climate change impact. Here, we demonstrate evidence of parallel evolution in adaptive tolerance to heat stress in dwarf cattle breeds (DCB, *Bos taurus indicus*) and compare their thermoregulatory responses with those in standard size cattle breeds (SCB, crossbred, *Bos taurus indicus *× *Bos taurus taurus*). We measured vital physiological, hematological, biochemical, and gene expression changes in DCB and SCB and compared the molecular phylogeny using mitochondrial genome (mitogenome) analysis. Our results show that SCB can acclimatize in the short term to higher temperatures but reach their tolerance limit under prevailing tropical conditions, while DCB is adapted to the warmer climate. Increased hemoglobin concentration, reduced cellular size, and smaller body size enhance thermal tolerance. Mitogenome analysis revealed that different lineages of DCB have evolved reduced size independently, as a parallel adaptation to heat stress. The results illustrate mechanistic ways of dwarfing, body size‐dependent tolerance, and differential fitness in a large mammal species under harsh field conditions, providing a background for comparing similar populations during global climate change. These demonstrate the value of studies combining functional, physiological, and evolutionary approaches to delineate adaptive potential and plasticity in domestic species. We thus highlight the value of locally adapted breeds as a reservoir of genetic variation contributing to the global domestic genetic resource pool that will become increasingly important for livestock production systems under a warming climate.

## 1 INTRODUCTION

Climate change is one of the key threats to agricultural production and indeed to the species survival in the Anthropocene (Godfray et al., 2010; Hoffmann, Sgrò, & Kristensen, 2017; Johnson et al., 2017). Body size constrains tolerance and the smaller size is an adaptation to the warmer climate (McCain & King, 2014; Pacifici et al., 2017; Savolainen, Lascoux, & Merila, 2013). Hence, how species cope with change will be important in defining their likelihood of future success (Mitchell et al., 2018; Pacifici et al., 2015). As we cannot predict all traits that may in future be advantageous, the conservation of biodiversity within domestic livestock is important, especially in the face of rapidly depleting biodiversity during climate change (Isbell et al., 2015; Mitchell et al., 2018). Moreover, heat stress is becoming an increasingly important constraint on animal productivity in various parts of the world (Collier, Renquist, & Xiao, 2017; Mitchell et al., 2018; Pacifici et al., 2015). Potential solutions for vulnerable populations are to engineer the environment (e.g., controlled environment buildings), which may prove to be unsustainable from an economic, environmental, or animal welfare perspective, or to change/adapt the animal to one that is more suited to the climate (Godfray et al., 2010; Hoffmann et al., 2017; Johnson et al., 2017). Here, we focus on the latter, which requires an understanding of the mechanisms of heat acclimatization and/or adaptation in animals (Pacifici et al., 2015, 2017 ; Savolainen et al., 2013; Seebacher, White, & Franklin, 2015). This includes a knowledge of the genetic architecture of traits, that is, how functional traits like heat tolerance and body size can change through individual phenotypic plasticity or population‐level evolution (Klockmann, Gunter, & Fischer, 2017; Pacifici et al., 2015, 2017 ). To date, few studies have attempted to analyze the differential tolerance to warming and underlying functional mutations among phenotypically disparate populations (Brans et al., 2017; Seebacher et al., 2015). Specifically, previous studies lack either a wide‐ranging understanding of the mechanism behind functional traits at physiological and molecular levels or the genetic milieu by which they evolved, or both (Pacifici et al., 2015, 2017 ).

As climate changes, organisms adapt, acclimatize, move, or die (Habary, Johansen, Nay, Steffensen, & Rummer, 2017). The differential tolerance may be due to the plasticity in populations facing opposing environmental conditions, as well as family‐specific innate plasticity that could enable adaptive variation (Savolainen et al., 2013; Seebacher et al., 2015). Adaptation to the environment is a complex and energetic continuing process caused by mutations arising and diffusing through populations (Savolainen et al., 2013), whereas acclimatization involves changes in physiology including through gene expressions (Pacifici et al., 2015; Seebacher et al., 2015). Specifically, temporal and spatial variations in traits like animal body size are explained as an adaptive response to climate warming and/or driven by changes in environmental productivity and food availability (Gardner, Peters, Kearney, Joseph, & Heinsohn, 2011; Martin, Mead, & Barboza, 2018). For instance, physiological acclimatization to environmental changes involves variation of the heat shock response, whereas other mechanisms mediate evolutionary changes in adaptive capability related to environmental gradients (Cahan et al., 2017). Gearty, McClain, and Payne (2018) demonstrated that body size changes and associated increased evolutionary rate are better explained using an energetic cost model, integrating size‐reliant functions for feeding and energy spending on metabolism and thermoregulation. Thus, spatial and temporal variations in climate drive current global patterns of biodiversity and determine local adaptation (Savolainen et al., 2013; Tilman et al., 2017). In addition, both physiological and energetic limitations can shape trait distributions (including body size) across climatic gradients (Classen, Steffan‐Dewenter, Kindeketa, & Peters, 2017).

The traits can evolve over a long period of time (Slater, 2015), but quickly as well (Geerts et al., 2015). Decline in body size is one of the universal responses to warming beside variations in phenology and dissemination (Gardner et al., 2011). In warmer climates, at physiological level, thermal stress response is mediated through the hypothalamo‐hypophyseal‐adrenal axis (Withers, Cooper, Maloney, Bozinovic, & Neto, 2016). Rectal temperature, respiratory rate, and heat tolerance index (HTC) that combine both rectal temperature and respiratory rate can predict breed differences in heat tolerance in humid tropical environments (Amakiri & Funsho, 1979; Charoensook et al., 2012; McManus et al., 2009). On the other hand, at the molecular level, two independent major stress response pathways are heat shock protein/heat shock factor (HSP/HSF) and reactive oxygen species (Gill et al., 2017). The HSP 70 is considered as a biomarker for heat stress in cattle (Mehla et al., 2014). The expression profiles of ATP1A1 (ATPase Na+/K+ Transporting Subunit Alpha 1, signaling gene involved in ion‐pumping), GAPDH (glyceraldehyde 3‐phosphate dehydrogenase, a gene related to energy metabolism), and ACTB (beta‐actin, a cytoskeletal actin) are also altered in cattle exposed to heat stress (Gill et al., 2017; Mehla et al., 2014). Mitochondria integrate environmental stimuli to modify gene expression patterns through mitonuclear communication and act as a controlling pivot in metabolism as well as during stress response (Harbauer, Zahedi, Sickmann, Pfanner, & Meisinger, 2014). In addition, mitochondrial diversity is also used to analyze the phylogenetic origin of breeds/populations (Liu, Cai, Liu, & Zhang, 2018; Marinov, Teofanova, Radoslavov, & Hristov, 2018). In domestic cattle, mitogenome analysis provides information regarding how functional traits like body size and tolerance are evolved in different lineages over temporal and spatial scales (Achilli et al., 2009, 2008 ). Thus, by simultaneously measuring both physiological and molecular responses, it is possible to assess differential tolerance among dissimilar genetic groups of animals (Albon et al., 2017; Alfonzo et al., 2016; Mitchell et al., 2018). Additionally, mitogenome analysis can map out the observed differential adaptive trait variations at physiological and molecular levels to the phylogeny of different genetic groups (Lajbner, Pnini, Camus, Miller, & Dowling, 2018).

**Figure 1:**
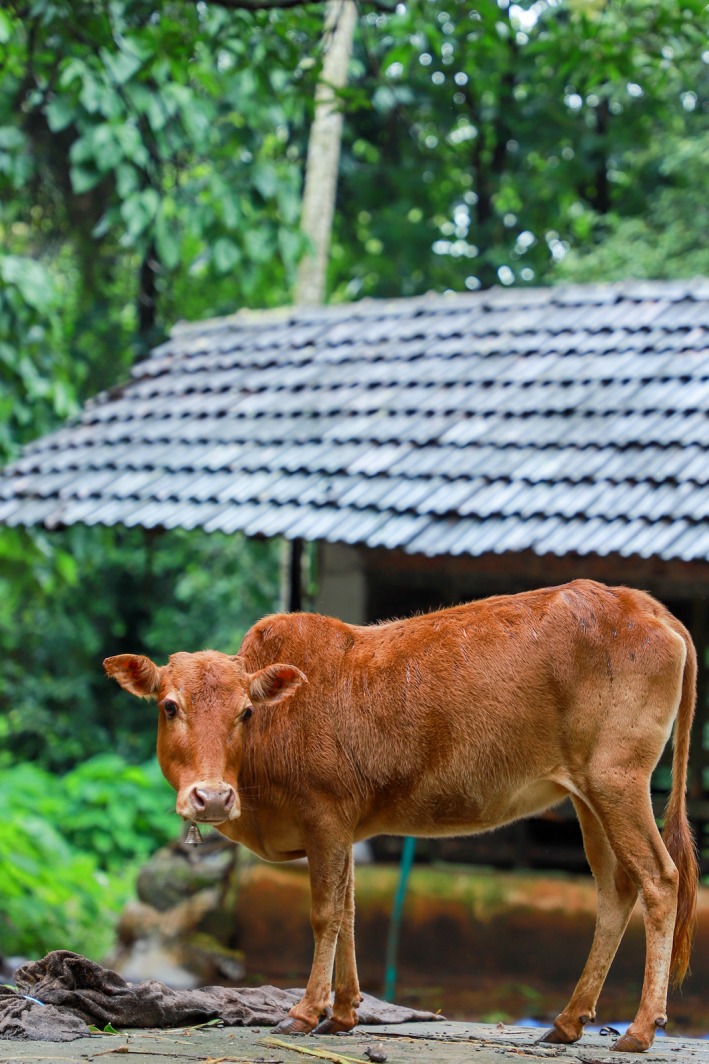
Manikyam—the smallest Vechur cattle (Guinness World Records Limited, 2016). Vechur is the smallest breed of cattle originated in the Vechur area in Kerala, India. The average weight and height of adult Vechur cattle range from 50 to 130 kg and 61 to 90 cm, respectively

Generally, separate and geographically isolated populations that are widely distributed and abundant enhance the ability to identify genetic architecture linked with phenotypic variations (Savolainen et al., 2013). In domestic cattle, genetic composition has been shaped by geographic segregation, ancient human movements, cross‐breeding, and gene flow among domestic and wild cattle populations (Jia et al., 2010; Park et al., 2015; Taye et al., 2017; Troy et al., 2001). Two subspecies of cattle; *Bos taurus indicus* (indicine) and *Bos taurus taurus* (taurine), were domesticated from extinct aurochs (*Bos primigenius*) (Decker et al., 2014). Indicine and taurine lineages are diverged from auroch ancestors about 0.74–1 Mya (Loftus, MacHugh, Bradley, Sharp, & Cunningham, 1994) and three main assemblages of modern cattle: African and Eurasian taurine and Asian indicine are now farmed (Upadhyay et al., 2017). Indicus haplotypes 1 and 2 diverged from a common ancestor about 5.3 ± 2.6 and 10.9 ± 3.5 thousand years ago, respectively (Hiendleder, Lewalski, & Janke, 2008). Asian indicine cattle are composed of *B. t. taurus*,* B. t. indicus*, and *B. javanicus* (Decker et al., 2014). The locally adapted hybrids in Asia, Africa, and America are crosses of hump‐less taurine and humped indicine (also called zebu) cattle, while the African taurine lineage predominates in European Mediterranean breeds (Decker et al., 2014). Hence, domestic cattle display extensive temporal and spatial variations in phenotype and genetic makeup (Elsik, Tellam, & Worley, 2009) including body size variation such as dwarfism, the molecular genetic basis of which also varies widely. For example, Boegheim, Leegwater, Lith, and Back (2017) explained that the inherited forms of dwarfism in some cattle breeds are caused by genetic mutations leading to structural, hormonal, and signaling pathway disruptions. However, DCB may be evolved following dispersal to extremely isolated environments, for example, the evolution of dwarf *Anoa* buffaloes at Sulawesi and Sunda islands (Rozzi, 2017). The Indicus haplotype consists of both dwarf (DCB) and standard size (SCB) cattle breeds. A proportionate reduction in body size (and hence a greater surface area to volume ratio to improve thermoregulation) is one possible evolutionary adaptation to increasing heat stress (Collier & Gebremedhin, 2015; Rozzi, 2017; Savolainen et al., 2013) but this is subject to evolutionary and agronomic trade‐offs (Tilman et al., 2017). For example, larger high‐yielding breeds are preferred over native smaller breeds in intensive agriculture. However, DCB such as the Vechur breed (Figure 1) may represent candidates for adaptation to global climate change due to their climatic resilience (Eisler et al., 2014). Kerala state in India, with 93% crossbred SCB (*B*. *t*. *indicus *× *B*. *t*.* taurus*) and 6% DCB (*B*. *t*. *indicus*), represents a large‐scale “natural experimental spot” for studying domestic cattle evolution in action.

Climate is one of the main explanatory variables for large ruminant morphology and largely, phenotypic changes are confined locally due to geographical isolation (Hill, Hill, & Widga, 2008; Martin et al., 2018). However, not all species have decreased in size over time and mechanisms other than improved heat dissipation may contribute to size reduction (e.g., changes in food availability or hunting; see Hill et al., 2008; Machac, Graham, & Storch, 2018). Increased temperature and humidity affect physiology and in turn functional traits like body size in different ways (Kim, Park, & Sin, 2018). There is an inverse relationship between enhancing environmental temperature and body size of ruminants in the last 40,000 years since warming decreases body size by altering metabolic loads and available resources (Martin et al., 2018). Phylogenetic diversity (PD, which measures evolutionary history among taxa) and functional diversity (FD, that represents quantitative measures of functional traits, like body size) capture the patterns in the diversity of traits, and studying their interaction can be informative (Tucker, Davies, Cadotte, & Pearse, 2018). Thus, linking physiology and phylogeny may help to identify mechanisms of dwarfing in cattle and aid to forecast the effect of environmental warming on ruminant adaptation and evolution. We hypothesized that concomitant increase in temperature and humidity in Kerala has resulted in adaptive changes in physiology and genetic architecture which may have facilitated a high level of morphological diversification in cattle, leading to the evolution of dwarf breeds.

In the present study, we assessed the acute heat tolerance in DCB (Vechur and Kasargode) and SCB (crossbreds) in a tropical field environment by measuring changes in both phenotypic and genotypic traits. Using a combined physiological and phylogenic approach, we explain how and why body size declined in domestic cattle and how this enhanced heat tolerance. Our aims were to understand the physiological basis and demonstrate the evolutionary origins of differential heat adaptation and/or acclimatization in morphologically distinct domestic cattle. Specifically, we evaluated the effects of acute heat stress, and rates of climatic‐niche evolution of functional traits, in a morphologically distinct population of domestic cattle. Next, we determined whether the variation in traits changed systematically across genetic groups representing different molecular mitochondrial phylogenetic scales by mapping evolutionary processes on to the trait diversity (Lajbner et al., 2018). Thus, we explored the potential for integrating physiological responses with molecular phylogeny to appreciate the physiological and evolutionary costs of body size changes.

## 2 MATERIALS AND METHODS

### 2.1 Study site

Kerala (10.8505°N, 76.2711°E), located in SW India, has hot and humid summer season from January to May and a warm and humid monsoon season from June to December. The average temperature humidity index (THI, see below) ranges from 72 to 83 throughout the year. Although the maximum temperature rarely rises above 35°C, relative humidity is high resulting in high THI, which can cause high heat stress in cattle. Dwarf cattle breeds (DCB) and standard size cattle breeds (SCB) are ideal for comparative studies because they represent closely related intraspecific incipient breeds, with vastly different size, which have radiated to fill different geographical niches (see Supporting information Figure S1).

### 2.2 Meteorological data and heat stress assessment

Temperature humidity index (THI, expressed as arbitrary unit) denotes the combined effect of ambient temperature and humidity, and is used to monitor heat stress impact in cattle. However, the THI does not comprise key climatic variables such as wind velocity and intensity of solar radiation. Similarly, THI does not account management factors (e.g., access to shade) or animal factors (genotype differences). Heat load index (HLI, expressed as arbitrary unit) is a measure of body heat gain and a correlated index, and the accumulated heat load (AHL, expressed as arbitrary unit) takes into account the duration of exposure to heat. The Accumulated Heat Load Index (AHLI, expressed as arbitrary unit) is the cumulative AHL over a given period in a day (Gaughan, Mader, Holt, & Lisle, 2008; Gaughan, Mader, Holt, Sullivan, & Hahn, 2010). These indices are used for assessing differential tolerance among purebred and crosses of *Bos taurus* and *Bos indicus* cattle (Lees, Lees, Lisle, Sullivan, & Gaughan, 2018) and also for developing genomic estimated breeding values (GEBV) for heat tolerance (Nguyen, Bowman, Haile‐Mariam, Pryce, & Hayes, 2016). The ambient temperature (Ta, °C), relative humidity (RH, %), intensity of solar radiation (SR, Wm^–2^), and wind speed (WS, ms^–1^) over the study period were obtained from nearby Kerala Agricultural University automatic weather station, and the THI, HLI, and AHL were determined.

THI = (1.8 × Ta + 32) – (0.55 – 0.0055 × RH)  ×  (1.8 × Ta – 26)
HLI = 8.62 + (0.38 × RH) + (1.55 Ta – 0.5 WS) + [e^2.4–WS^]

AHL = IF [HLI < HLILT, (HLI–HLILT)/M], IF [HLI > HLIUT, (HLI–HLIUT)/M, 0)], where HLILT is the HLI threshold below which cattle will dissipate heat (here, 81 for crossbred cattle), HLIUT is the HLI threshold above which cattle will gain heat (here, 90 for crossbred cattle), and M the number of measurements per hour (here *M* = 2, Bohmanova, Misztal, & Cole, 2007).

These indices vary in their ability to evaluate heat stress. For example, for humid climates, indices with a larger weighting for humidity are used (Bohmanova et al., 2007). Generally, the lower threshold THI value for cattle is 72 (Bohmanova, Misztal, Tsuruta, Norman, & Lawlor, 2008). For purebred and crosses of *Bos taurus* cattle, HLI from 70 to 96 indicates thermoneutral to extreme heat load conditions (Gaughan, et al., 2010).

### 2.3 Animals

The Vechur and Kasargode cattle were derived from local populations in the Vechur and Kasargode areas of Kerala and conserved ex situ at the Kerala Veterinary and Animal Sciences University farm. Ten adult lactating nonpregnant animals each of Vechur, Kasargode, and SCB (30 in total) were selected from the wider population at the university farm. The selected animals grazed native pasture during the day and were housed at night. They were trained in the sampling procedures in a presampling exposure period, which reduced handling stress during the sampling period. The animals were free of any infectious diseases and were all in good health. Over a period of 10 days in the summer, we observed the animals, grazing from 08.00 to 14.00 under THI ranging from 75 to 83. After morning milking, grass ration (Napier grass; *Pennisetum purpureum*, harvested the same day) and drinking water were given. Baseline physiological measurements were made, and a blood sample collected in the shed before cattle was taken to the pasture at 08:00, where they grazed with no shade until 14.00. Drinking water was provided ad libitum. The Institutional Animal Ethics Committee of Kerala Veterinary and Animal Sciences University, Kerala, India, had approved the experimental protocol.

### 2.4 Response variables

#### 2.4.1 Physiological measurements

We measured physiological variables at half‐hour intervals starting from 08:00 to 14:00. We recorded rectal temperature (RT, °C.) with a digital clinical thermometer. We counted flank movements for 1 min with the help of a stopwatch and recorded respiration rate (RR, breaths/min). The heat tolerance coefficient (HTC, expressed as arbitrary unit) was derived from physiological measurements, HTC = RR/23 + RT/38.3 (Bianca, 1963). Pulse rate (PR, beats/min) was recorded for 1 min using a stethoscope. Starting from 08:00, 5 ml of blood was collected at 2‐hr intervals via the jugular venous puncture in the vacutainer with 5 mg EDTA as the anticoagulant, under aseptic conditions for hematological and genetic analysis. Blood samples (5 ml) without EDTA were also collected, centrifuged at 450 × *g* for 10 min, and stored at −20°C for determination of serum cortisol concentration using an enzyme immunoassay kit (EIA steroid cortisol kit, Agappe Diagnostics Limited, India).

#### 2.4.2 Quantitative real‐time PCR (Q‐RT‐PCR)

We isolated total l RNA immediately after collecting blood using GeneiPure RNA extraction kit (Cat. No. KT‐173, Genei, Bangalore) following the manufacturer's recommendations. All solutions and buffers were prepared in RNase‐free glassware and 0.1% DEPC (diethylpyrocarbonate)‐treated water. Before beginning the experiment, consumables, equipment, and work surfaces were made RNase‐free by using RNaseZAP^®^ solution (Cat. No. R2020, Sigma‐Aldrich). To make blood samples RNase‐free, RNAlater^®^ (Cat. No. R0901, Sigma‐Aldrich) was used. DNase treatment was conducted using DNase1 kit (Cat. No. AMP‐D1, Sigma‐Aldrich). The RNA was quantified using a spectrophotometer (NanoDrop ND‐1000, Thermo Scientific, USA). We checked the RNA quality using agarose gel electrophoresis (0.8%). The relative quantification of gene expression was carried out using Illumina Eco® Q‐RT‐PCR system using SYBR green chemistry, giving the difference in expression (ΔCt) of target genes HSP70 (heat shock protein 70), ATP1A1 (sodium potassium ATPase), and GAPDH (glyceraldehyde 3 phosphate dehydrogenase) versus reference gene ACTB (Sambrook, Fritsch, & Maniatis, 1989). The GAPDH was used as reference gene for ACTB. After exposure to heat stress, the fold changes (relative quantification—RQ) in the expression of the above four genes were assessed by comparing between genetic groups using RQ = 2^–∆∆Ct^. The oligonucleotide primers for HSP70, ATP1A1, ACTB, and GAPDH genes were designed using IDT primer design software (http://www.idtdna.com/Primerquest) and custom synthesized from Sigma‐Aldrich (Supporting information Table S1). The cDNA was synthesized from a constant amount (1 µg) of total RNA using cDNA synthesis kit (Cat. No. K1621, Fermentas) as per the manufacturer's instruction. For Q‐RT‐PCR, Maxima SYBR Green Q‐PCR Master Mix with ROX was used (Cat. No. K0221, Thermo Scientific) and was carried out in 96‐well plates in a thermal cycler (Bio‐Rad, Thermal cycler, USA) as per manufacturer's instructions. Separate PCR reactions were set up for HSP70, ATP1A1, ACTB, and GAPDH genes. We amplified each sample in triplicate (technical replicates) in a reaction volume of 12.5 µl, which contained 1 µl of cDNA+1 µl each of forward and reverse primers (10 pm/µl) + 6.25 µl Maxima SYBR Green qPCR Master Mix (2X) + 3.25 µl nuclease‐free water. We followed two‐step Q‐RT‐PCR protocol. The segment 1comprised of enzyme activation (single cycle, 95°C for 10 min). Segment 2 included denaturation and annealing/extension (35 cycles, 95°C for 15 s, and 60°C for 60 s, respectively). These were followed by a melting step by gentle heating from 62°C to 75°C and finally a cooling down at 4°C. We performed data acquisition during the annealing step. In addition, one nontemplate control (NTC) for each gene and reverse transcription minus (RT minus) control for each sample and a negative control (with only nuclease‐free water) were also included.

#### 2.4.3 Mitochondrial genome sequencing

We sequenced a set of four mitochondrial DNA (mtDNA)—three dwarf cattle; Vechur, Kasargode, and Wayanad, and one crossbred cattle by using long‐range PCR. We amplified the entire mtDNA genome with a set of two overlapping PCR fragments. Sequencing was done by next‐generation sequencing with Illumina HiSeq. (Primers‐F15'TTAACCCAAAGCAAGGCACT3, R15'TGAGGATTGTTAGGGCTGCT 3', F25'CCAAGCCTATGTATTCACTCTCC3', R2 5'GGGGCCTGCGT TTA TATA TT G3'). The amplicons were fragmented, end repaired, adenylated, adapter‐ligated, and then amplified by PCR. The amplified DNA library was run on the tape station for size distribution, and the concentration was measured using Qubit. The DNA library thus prepared was sequenced on the HiSeq that generated 2 × 250 bp paired‐end reads. Quality testing like base quality score distribution, sequence quality score distribution, and GC distribution were done, and quality sequences were retained for further analysis. Illumina adapters were trimmed from paired‐end reads using CutAdapt (Martin, 2011). The paired‐end reads were assembled using IVA (Hunt et al., 2015) and checked for errors using SEQuel (Ronen, Boucher, Chitsaz, & Pevzner, 2012). The assembled mitochondrial genomes were annotated using MITOS (Bernt et al., 2013), and the diagrammatic representation of the mitochondrial genomes was created using CGView (Stothard & Wishart, 2004).

#### 2.4.4 Statistical analysis

We conducted statistical tests in R version 3.5.1 (R Core Team, 2018). We tested the impact of heat stress on different breeds (physiological, hematological, serum cortisol, and relative expression of candidate genes) using separate linear mixed‐effect models. For model fitting, we used the “lme4” package. The correlation plots and principal component analyses were done using “ggplot2, devtools, and ggbiplot” packages. We tested the influence of body size and temperature humidity index (THI) on acute heat stress response of cattle with repeated‐measures ANOVA using linear mixed‐effect models and report the final accepted model. Animals were taken as the random effect. ANOVA (model) in “car” package was used. The random effects in the model were checked using “gls” function in “nlme” package. We selected *p*‐value and pseudo‐*R*‐squared for the model using the “nagelkerke” function in “rcompanion” package. Post hoc analysis was done using Tukey adjusted comparisons in “lsmeans and multcompview” packages. Interaction plots were made using “groupwiseMean” function in “rcompanion and ggplot 2” packages. We estimated the natural means of each breed with THI grouping. We calculated confidence interval of each means with the percentile method and checked for homoscedasticity and independence by plotting residuals versus fitted values.

#### 2.4.5 Phylogeny

We linearized the mitochondrial genomes from the 12S rRNA gene and aligned in MAFFT v.7.308 (Katoh & Standley, 2013; Katoh, Misawa, Kuma, & Miyata, 2002) in Geneious 10.0.9 (Kearse et al., 2012). For phylogenetic reconstruction, we used the GTR + I + G nucleotide substitution model for the preliminary phylogeny, and further the HKY + I + G model to fine‐scale *Bos indicus* phylogeny, using AIC in jModeltest 2.1.10 (Darriba, Taboada, Doallo, & Posada, 2012; Posada, 2008). The maximum‐likelihood phylogeny was reconstructed using PhyML (Guindon & Gascuel, 2003). The substitution model was selected in jModeltest; beginning the tree with optimized topology, length, and rate factors. Topology searching was done by the best of NNI and SPR, using 500 bootstraps. Origin and subhaplotype affiliation of mitogenomes considered in this study are given in Supporting information Table S5.

## 3 RESULTS

### 3.1 Physiological responses

**Figure 2:**
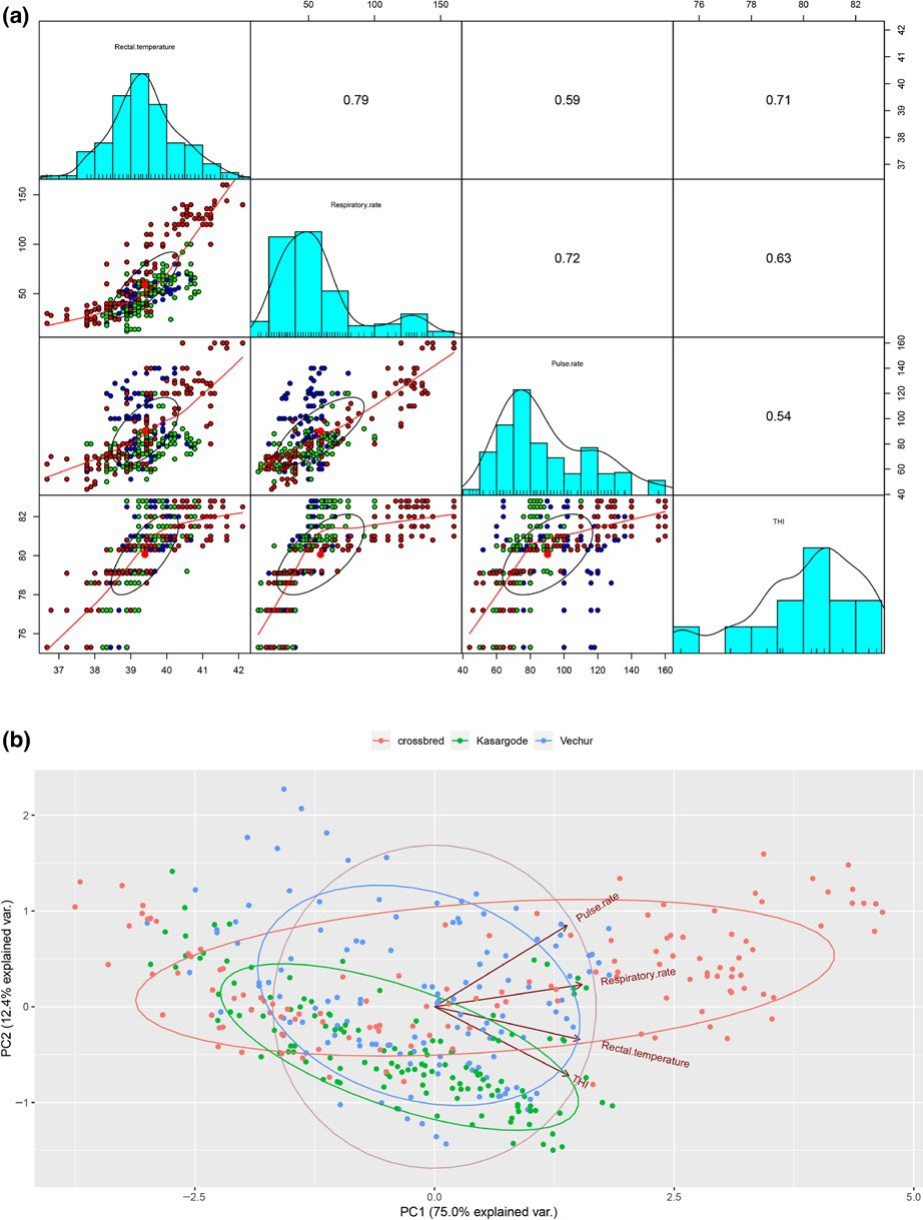
Changes in vital physiological parameters in response to heat stress (a) Correlation plot showing changes in rectal temperature ‐°C (RT), respiratory rate‐breaths/minute (RR), and pulse rate‐beats/minute (PR) in response to increase in temperature humidity index (THI) from 75.3 to 82.8 under field conditions in Vechur (blue), Kasargode (green), and crossbred (red) cattle. (b) Principal component analysis biplot showing the relationship of RT, RR, and PR with THI. Endotherms maintain constant RT by altering the physiological parameters—RR and PR. Here, heat‐sensitive crossbred cattle shows heterothermy. Vechur and Kasargode cattle employ differential thermoregulatory strategies as Vechur relied more on PR, while Kasargode relied on RR. Molecular phylogeny of different genetic groups using mitochondrial genome sequencing explains evolutionary sequel of this phenomenon

We calculated mean temperature humidity index (THI) for different periods of the day: prestress (08:00–10:00, THI = 75.3–79.2) and heat stress (10.00–14:00, THI = 80.5–82.8, Supporting information Figure S2). For SCB, a threshold HLI of 90 was observed, whereas for DCB the threshold HLI was not reached. Accumulated heat load (AHL, a measure of heat load) was evident in SCB but not in DCB. Under increasing AHL, SCB showed clinical signs of thermal strain such as open‐mouthed breathing, salivating, reluctance to rise, enhanced licking of the skin, and overall dullness. SCB had a low rectal temperature (RT) relative to DCB at the beginning of the study, but this increased upon heat exposure (Figure 2, Supporting information Tables S2–S4, Supporting information Figure S3). In addition, SCB had RT_diff_ (difference in rectal temperature before and after heat exposure) of 5.4°C, while that for Vechur and Kasargode, cattle was 2.9°C and 3.1°C, respectively. Trends for RT and respiratory rate (RR) were similar in Vechur and Kasargode but trends for pulse rate differed between them (Figure 2, Supporting information Tables S2–S4, Supporting information Figures S3–S5). Heat tolerance coefficient (HTC, a measure of heat tolerance) combining RT and RR proved to be a good indicator of tolerance in different genetic groups studied (Supporting information Figure S6).

### 3.2 Hematology, serum cortisol, and gene expression

**Figure 3:**
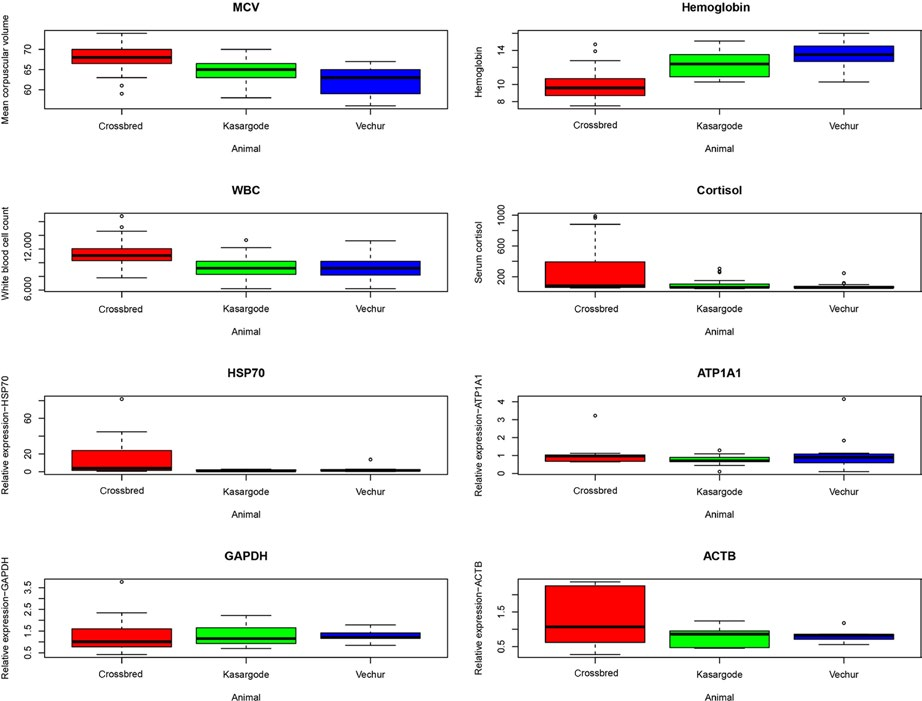
The effect of the increase in temperature humidity index (THI) from 75.3 to 82.8 under field conditions in Vechur, Kasargode, and crossbred cattle on hematological, serum cortisol, and gene expression. We propose the change in MCV as one of the mechanisms of dwarfing in Vechur and Kasargode cattle. Increased hemoglobin concentration in dwarf cattle shows their high tolerance. White blood cell count, cortisol, and relative expression (RE) of HSP70 gene showed a similar pattern in crossbred cattle. The RE of ATP1A1, GAPDH, and ACTB genes showed similar trends in both dwarf and standard size breeds

Among hematological parameters, DCB had low mean corpuscular volume (MCV), the typical volume of a red blood cell (Figure 3, Supporting information Table S3, Supporting information Figure S7). Hemoglobin concentration increased as body size reduced (Figure 3, Supporting information Table S3, Supporting information Figures S8–S9). Other hematological values such as red blood cell count, white blood cell count, neutrophil–lymphocyte ratio, and packed cell volume were also altered in SCB (Figure 3, Supporting information Table S3, Supporting information Figures S10–S13). Heat stress triggered significant cortisol secretion, and upregulation of HSP70, but not the other nonheat shock response (non‐HSR) candidate genes (Figure 3, Supporting information Table S3, Supporting information Figure S14).

### 3.3 Phylogeny

**Figure 4:**
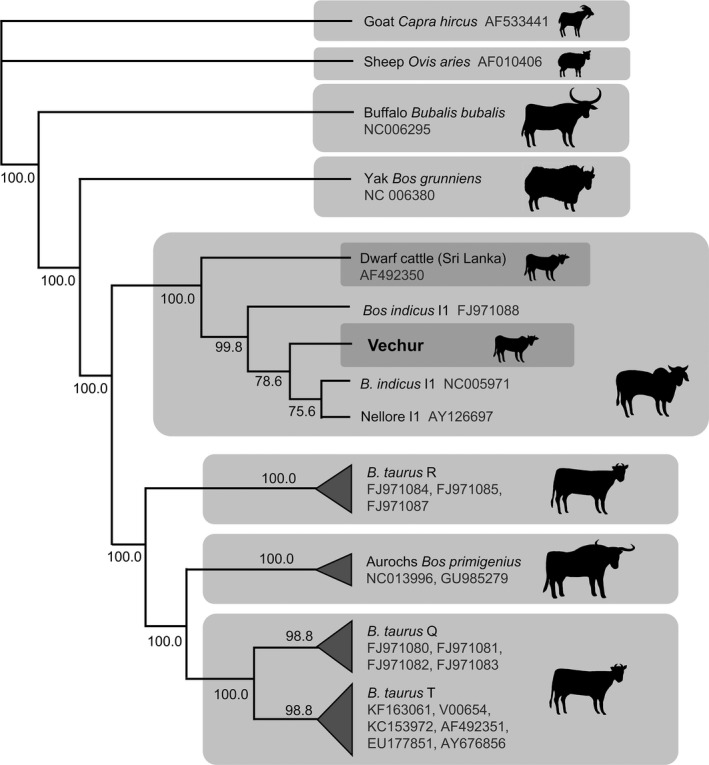
Maximum‐likelihood cladogram showing the position of the Vechur dwarf cattle within the *Bos* genus, with sheep and goat as outgroups. Branch lengths are in substitutions/site. Clade support values are % bootstrap support from 500 runs; nodes with fewer than 50% support have been collapsed. For further details of sequences used, see Supporting information Table S5

**Figure 5:**
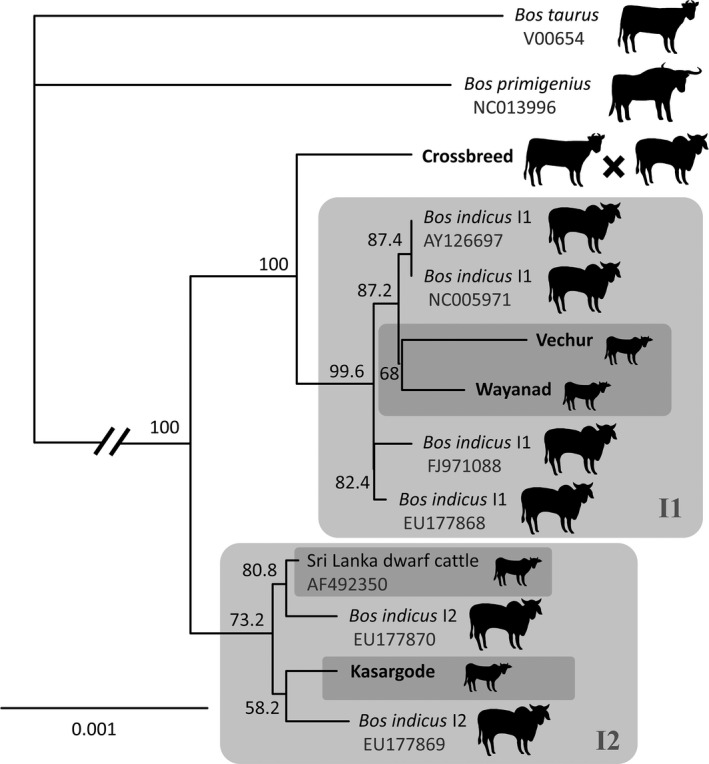
Maximum‐likelihood phylogram of *Bos indicus*, with *B. taurus* reference sequence as the outgroup. Branch lengths are in substitutions/site. Clade support values are % bootstrap support from 500 runs. Lineages in bold were sequenced in the current study; GenBank accessions are shown for all (see Supporting information Table S5)

We used the mitogenome of the world's smallest cattle (Vechur breed, Guinness World Records Limited, 2016) and other *B. taurus* and *B. indicus* cattle to reconstruct the maximum‐likelihood phylogeny, with sheep and goat sequences as the outgroup (Supporting information Table S5). As expected, Vechur fell into the *B. indicus* clade (Figure 4). A maximum‐likelihood phylogeny was then reconstructed for breeds within the *B. indicus* group, including other DCB and a crossbred lineage, with the *B. taurus* reference sequence as the outgroup. Vechur and Wayanad, other dwarf cattle from a different area of Kerala, India, clustered in the Indicus 1 haplotype (I1), while Kasargode clustered in the Indicus 2 (I2) haplotype, more closely related to SCB I2 than the other DCB (Figure 5).

## 4 DISCUSSION

Our data support our hypothesis that warm climate has caused a decline in the body size of domestic cattle *Bos* spp., as DCB is tolerant and adapted. However, SCB has acclimatized to heat through physiological plasticity, reflecting the parallel evolution of adaptive thermal tolerance and body size. Specifically, the adaptation in dwarf cows has been mediated through evolutionary changes as evidenced by molecular phylogenetic analysis using the mitochondrial genome, while acclimatization in crossbred cows has been achieved through alterations in physiological, hormonal, and gene expression profiles. We also observed that genetic changes in the mitochondrial genome are associated with cellular and body size and hemoglobin concentration in phenotypically disparate cattle breeds that are evolved in different geographical niches. These findings illustrate that reduction in body size increases heat tolerance. Our study delineates the adaptive and plastic phenotypic and genotypic changes, both in body size and in thermal tolerance in domestic cattle shaped by parallel evolution and acclimatization, and improves our understanding of species responses to climate warming.

Homeostatic regulators of physiological, hematological, endocrine, and molecular pathways drive the acute heat stress response, which in turn is modified by the genetic architecture (Mitchell et al., 2018). Cattle rely more on evaporative cooling for heat dissipation (Mitchell et al., 2018), with humidity as the limiting factor in humid climates and temperature in dry climates (Bohmanova et al., 2007). The body weight and height of adult DCB range from 50 to 130 kg and 61 to 90 cm, respectively. To meet the growing demand for milk and meat, crossbred cattle (SCB), which are crosses of taurine breeds (Holstein‐Friesian, Jersey, and Brown Swiss) with standard‐sized indicine/zebu breeds (mostly Red Sindhi), were introduced to Kerala in the 1960 s. They now account for approximately 93% of the cattle population in Kerala. The average height and weight of SCB are 120–150 cm and 300–375 kg, respectively. Vechur has a high genetic distance from other Indian DCB such as Malanad Gidda and Punganur (Ramesha et al., 2016). A decline in body size is considered consistent with warming (Gardner et al., 2011; Klockmann et al., 2017) as smaller individuals are better able to dissipate heat (Martin et al., 2018). As observed in our study, under high humidity SCB cannot exchange heat optimally, resulting in heat accumulation. The dynamics of climate driven diversification and distribution change with the growth and accumulation of clades over time at different locations (Machac et al., 2018). Thus, the positive heat balance in SCB over an evolutionary period may have caused genetic changes resulting in dose‐dependent variation in traits including body size, eventually resulting in the origin of dwarf cattle in hot and humid southern coastal areas of the Indian subcontinent (Martin et al., 2018; Rozzi, 2017).

One notable feature observed in the study was the difference in vital physiological and hematological parameters between Vechur and Kasargode during heat stress. In Vechur, the slope of the relationship between rectal temperature and pulse rate was greater than for Kasargode, which had a stronger relationship between rectal temperature and respiratory rate. Specifically, Vechur relied more on pulse rate, while Kasargode relied on the respiratory rate to maintain body temperature. This differential degree of dependence was also evident in other response variables. The differential strategy used by these two DCB lineages indicates that heat tolerance is not only associated with morphological characteristics like body size. This observation is also intriguing because it reveals that the two breeds from similar environments and with apparent phenotypic similarities employ different physiological pathways and thermoregulatory strategies. A previous study reported a predominance of sweating in some cattle breeds, while others were more prone to thermal polypnea during heat stress (Pereiraet al., 2014). Overall, Vechur was the most tolerant to heat stress, followed by Kasargode and SCB the least tolerant, and phylogenetic analysis revealed an independent parallel selection for this in the two DCB lineages. Therefore, by combining functional and evolutionary studies, we can conclude that both DCB lineages provide different sources of adaptive potential for resilience to climate change in livestock (Savolainen et al., 2013; Seebacher et al., 2015). The different phylogenetic origins and physiological tolerance mechanisms of co‐occurring, phenotypically similar populations highlight the importance of preserving domestic genetic diversity, including multiple local breeds with superficially similar adaptations, to maintain adaptive potential and future‐proof our domestic gene pools against environmental change (Hoffmann et al., 2017).

Also, one of the mechanisms of enhanced tolerance is increased hemoglobin concentration (Brans et al., 2017). Here, in DCB, hemoglobin concentration increased as body size reduced. In SCB, other hematological values including red blood cell count, white blood cell count, neutrophil–lymphocyte ratio, and packed cell volume were also found to be valid indicators of individual stress load. Further, two mechanisms for size reduction are reduced cell size and reduced cell number (Hessen, Daufresne, & Leinaas, 2013). We found that RBC is significantly smaller in DCB as evidenced by reduced MCV. Thus, breeds of different sizes may have evolved in different niches which may, in turn, have determined their thermoregulatory patterns (Pereira et al., 2014). We illustrate that, continued genetic changes selected under heat stress would have resulted in reduced cell volume and subsequent body size reduction in DCB (Gutierrez‐Alonso, Hawkins, Cools, Shaw, & Fraaije, 2017; Rabouille & Alberti, 2017).

Furthermore, body temperature variation is associated with reduced fitness, with more extreme daily fluctuations correlated with reduced reproduction in wild mammal populations (Maloney, Marsh, McLeod, & Fuller, 2017). Cells recognize environmental fluctuations through sophisticated signaling pathways and hence stress directly affects the cellular integrity, function, and morphology (Rabouille & Alberti, 2017) and shapes mitochondrial genome evolution (Lajbner et al., 2018). The maintenance of homeothermy during heat stress in SCB was achieved primarily by heterothermy. In SCB, this narrow physiological tolerance can result in an accumulated metabolic cost of plasticity and subsequent low fitness (Maloney et al., 2017). For instance, as high hemoglobin concentration is associated with a high metabolic rate at higher temperatures, driven by high oxygen demand (Portner & Knust, 2007), the low hemoglobin level in SCB reflects low protection against oxidative damage, making them more vulnerable to stress (Collier et al., 2017). Likewise, a significantly higher respiratory rate in SCB than DCB throughout the period of the study suggests total body deficit of bicarbonate (HCO_3_–), leading to respiratory alkalosis and the potential of subsequent metabolic acidosis and further stress susceptibility (Collier et al., 2017).

The phylogeny revealed that Vechur and Wayanad clustered in the Indicus 1 haplotype (I1), while Kasargode clustered in the Indicus 2 (I2) haplotype, showing a convergent evolution of dwarf size in response to high heat and humidity in cattle breeds in different regions (Taye et al., 2017). The mitochondrial genome of dwarf cattle might have evolved through selection under heat stress (Lajbner et al., 2018). In domestic cattle, dwarfing and tolerance is evolutionarily defined by functional traits developed through maternal founder effect and adaptation to warm environments (Lenstra et al., 2014). Moreover, dwarf cattle breeds—Vechur, Punganur and Malanad Gidda—follow a continuous distribution in the southern part of the Indian subcontinent. Specifically, zebu cattle (*Bos taurus indicus*) are a subset of taurine cattle (*Bos taurus taurus*), and dwarf cattle are a subset of zebu cattle—a serial multiple founder effects (Horsburgh et al., 2013). In summary, in the absence of gene flow, the isolated dwarf cattle populations might have adapted independently to their environment (Rozzi, 2017) through dose‐dependent selection (Gutierrez‐Alonso et al., 2017).

The current challenge is to understand adaptive capacity in different populations, using correlative, mechanistic, and trait‐based vulnerability assessments, particularly for those approaching physiological limits (Mazel, Mooers, Riva, & Pennell, 2017). For the first time, heat‐tolerant DCB are characterized at physiological, molecular, and phylogenetic levels. Previous studies have shown that heat stress responses encompass a complex network of pathways, even at the cellular level (Collier et al., 2017; Rabouille & Alberti, 2017; Savolainen et al., 2013). We describe heat stress response quantitatively, differentiating adaptive and plastic changes in response to temperature increase, and revealing different thermoregulatory strategies in different breeds of dwarf cattle. The results illustrate different physiological factors contributing to thermal limits of a species in a dose‐dependent manner (Gutierrez‐Alonso et al., 2017) and their capacity to cope with varying microclimates (Maloney et al., 2017).

The key strength of our work is the simultaneous evaluation of physiological, hormonal, and molecular changes along with molecular phylogenetic analysis using mitochondrial genomes of the different genetic groups studied. This could be supplemented with a broader study across morphologically disparate populations of livestock to assess how environment has influenced trait variations across different temporal and spatial scales. We also emphasize the importance of local responses to small‐scale environmental changes as a contributor to trait variations.

Our findings on body size and stress response variation based on evolutionary and physiological responses are likely to have wider applications for other wild and domestic species and offer insights into stress assessments to predict biological responses to global climate change (Collier et al., 2017; Maloney et al., 2017). In addition, changes in functional traits like body size have significant repercussions for the thermal biology and energetics of ruminants, as body size directly affects energy requisite for maintenance, growth, and production (Mitchell et al., 2018). We argue that variations in the body size of domestic cattle will, therefore, influence resilience to environmental change (Martin et al., 2018). Hence, a genomic, transcriptomic, proteomic, and metabolomic approach is needed to understand the underlying phenomena of body size‐related adaptability and acclimatization in diverse populations. Further studies using our combined physiological and molecular approach may elucidate further mechanistic differences between stress responses in other breeds and species that may assist to prioritize targeted interventions both to increase species resilience and their adaptive capability (Savolainen et al., 2013; Seebacher et al., 2015). To conclude, we must, therefore, select and breed carefully for sustainable livestock production and preserve the domestic genetic resource diversity we already have, as they may hold the solutions to adapt to climate change.

## CONFLICT OF INTEREST

The authors declare no competing interests.

## AUTHOR CONTRIBUTIONS

MEM led the research and carried out the analysis with ATV, MSK, and VS; NH advised on phylogenetic analysis; and MSK, VS, NH, THM, MJR, and MRFL contributed to the writing of the paper and the interpretation of the results.

## DATA ACCESSIBILITY

DNA sequences: GenBank accessions MF667929‐MF667932.

5

## References

[ece34550-bib-0001] Achilli, A. , Bonfiglio, S. , Olivieri, A. , Malusa, A. , Pala, M. , Kashani, B. H. , … Bandelt, H. J. (2009). The multifaceted origin of taurine cattle reflected by the mitochondrial genome. PloS One, 4(6), e5753 10.1371/journal.pone.0005753 19484124 PMC2684589

[ece34550-bib-0002] Achilli, A. , Olivieri, A. , Pellecchia, M. , Uboldi, C. , Colli, L. , Al‐Zahery, N. , … Battaglia, V. (2008). Mitochondrial genomes of extinct aurochs survive in domestic cattle. Current Biology, 18(4), R157–R158. 10.1016/j.cub.2008.01.019 18302915

[ece34550-bib-0003] Albon, S. D. , Irvine, R. J. , Halvorsen, O. , Langvatn, R. , Loe, L. E. , Ropstad, E. , … Hansen, B. B. (2017). Contrasting effects of summer and winter warming on body mass explain population dynamics in a food‐limited Arctic herbivore. Global Change Biology, 23(4), 1374–1389. 10.1111/gcb.13435 27426229

[ece34550-bib-0004] Alfonzo, E. P. M. , da Silva, M. V. G. B. , dos Santos Daltro, D. , Stumpf, M. T. , Dalcin, V. C. , Kolling, G. , … McManus, C. M. (2016). Relationship between physical attributes and heat stress in dairy cattle from different genetic groups. International Journal of Biometeorology, 60(2), 245–253. 26062817 10.1007/s00484-015-1021-y

[ece34550-bib-0005] Amakiri, S. F. , & Funsho, O. N. (1979). Studies of rectal temperature, respiratory rates and heat tolerance in cattle in the humid tropics. Animal Science, 28(3), 329–335. 10.1017/S0003356100023230

[ece34550-bib-0006] Bernt, M. , Donath, A. , Jühling, F. , Externbrink, F. , Florentz, C. , Fritzsch, G. , … Stadler, P. F. (2013). MITOS: Improved de novo metazoan mitochondrial genome annotation. Molecular Phylogenetics and Evolution, 69(2), 313–319. 10.1016/j.ympev.2012.08.023 22982435

[ece34550-bib-0007] Bianca, W. (1963). Rectal temperature and respiratory rate as indicators of heat tolerance in cattle. The Journal of Agricultural Science, 60(1), 113–120. 10.1017/S0021859600015902

[ece34550-bib-0008] Boegheim, I. J. , Leegwater, P. A. , van Lith, H. A. , & Back, W. (2017). Current insights into the molecular genetic basis of dwarfism in livestock. The Veterinary Journal, 224, 64–75. 10.1016/j.tvjl.2017.05.014 28697878

[ece34550-bib-0009] Bohmanova, J. , Misztal, I. , & Cole, J. B. (2007). Temperature‐humidity indices as indicators of milk production losses due to heat stress. Journal of Dairy Science, 90(4), 1947–1956. 10.3168/jds.2006-513 17369235

[ece34550-bib-0010] Bohmanova, J. , Misztal, I. , Tsuruta, S. , Norman, H. D. , & Lawlor, T. J. (2008). Genotype by environment interaction due to heat stress. Journal of Dairy Science, 91(2), 840–846. 18218772 10.3168/jds.2006-142

[ece34550-bib-0011] Brans, K. I. , Jansen, M. , Vanoverbeke, J. , Tuzun, N. , Stoks, R. , & De Meester, L. (2017). The heat is on: Genetic adaptation to urbanization mediated by thermal tolerance and body size. Global Change Biology, 23(12), 5218–5227. 10.1111/gcb.13784 28614592

[ece34550-bib-0012] Cahan, S. H. , Nguyen, A. D. , Stanton‐Geddes, J. , Penick, C. A. , Hernáiz‐Hernández, Y. , DeMarco, B. B. , & Gotelli, N. J. (2017). Modulation of the heat shock response is associated with acclimation to novel temperatures but not adaptation to climatic variation in the ants *Aphaenogaster picea* and *A*. *rudis* . Comparative Biochemistry and Physiology Part A: Molecular & Integrative Physiology, 204, 113–120. 10.1016/j.cbpa.2016.11.017 27894884

[ece34550-bib-0013] Charoensook, R. , Gatphayak, K. , Sharifi, A. R. , Chaisongkram, C. , Brenig, B. , & Knorr, C. (2012). Polymorphisms in the bovine HSP90AB1 gene are associated with heat tolerance in Thai indigenous cattle. Tropical Animal Health and Production, 44(4), 921–928. 10.1007/s11250-011-9989-8 22008953 PMC3289787

[ece34550-bib-0014] Classen, A. , Steffan‐Dewenter, I. , Kindeketa, W. J. , & Peters, M. K. (2017). Integrating intraspecific variation in community ecology unifies theories on body size shifts along climatic gradients. Functional Ecology, 31(3), 768–777. 10.1111/1365-2435.12786

[ece34550-bib-0015] Collier, R. J. , & Gebremedhin, K. G. (2015). Thermal biology of domestic animals. Annual Review of Animal Biosciences, 3(1), 513–532. 10.1146/annurev-animal-022114-110659 25387108

[ece34550-bib-0016] Collier, R. J. , Renquist, B. J. , & Xiao, Y. (2017). A 100‐Year Review: Stress physiology including heat stress. Journal of Dairy Science, 100(12), 10367–10380. 10.3168/jds.2017-13676 29153170

[ece34550-bib-0017] Darriba, D. , Taboada, G. L. , Doallo, R. , & Posada, D. (2012). jModelTest 2: More models, new heuristics and parallel computing. Nature Methods, 9(8), 772 10.1038/nmeth.2109 PMC4594756 22847109

[ece34550-bib-0018] Decker, J. E. , McKay, S. D. , Rolf, M. M. , Kim, J. , Alcalá, A. M. , Sonstegard, T. S. , … Babar, M. E. (2014). Worldwide patterns of ancestry, divergence, and admixture in domesticated cattle. PLoS Genetics, 10(3), e1004254 10.1371/journal.pgen.1004254 24675901 PMC3967955

[ece34550-bib-0019] Eisler, M. C. , Lee, M. R. , Tarlton, J. F. , Martin, G. B. , Beddington, J. , Dungait, J. A. , … Misselbrook, T. (2014). Agriculture: Steps to sustainable livestock. Nature, 507(7490), 32 10.1038/507032a 24605375

[ece34550-bib-0020] Elsik, C. G. , Tellam, R. L. , & Worley, K. C. (2009). The genome sequence of taurine cattle: A window to ruminant biology and evolution. Science, 324(5926), 522–528. 19390049 10.1126/science.1169588 PMC2943200

[ece34550-bib-0021] Gardner, J. L. , Peters, A. , Kearney, M. R. , Joseph, L. , & Heinsohn, R. (2011). Declining body size: A third universal response to warming? Trends in Ecology & Evolution, 26(6), 285–291. 10.1016/j.tree.2011.03.005 21470708

[ece34550-bib-0022] Gaughan, J. B. , Mader, T. L. , Holt, S. M. , & Lisle, A. (2008). A new heat load index for feedlot cattle. Journal of Animal Science, 86, 226–234. 17911236 10.2527/jas.2007-0305

[ece34550-bib-0023] Gaughan, J. B. , Mader, T. L. , Holt, S. M. , Sullivan, M. L. , & Hahn, G. L. (2010). Assessing the heat tolerance of 17 beef cattle genotypes. International Journal of Biometeorology, 54(6), 617–627. 10.1007/s00484-009-0233-4 19458966

[ece34550-bib-0024] Gearty, W. , McClain, C. R. , & Payne, J. L. (2018). Energetic tradeoffs control the size distribution of aquatic mammals. Proceedings of the National Academy of Sciences of USA, 115(16), 4194–4199. 10.1073/pnas.1712629115 PMC5910812 29581289

[ece34550-bib-0025] Geerts, A. N. , Vanoverbeke, J. , Vanschoenwinkel, B. , Van Doorslaer, W. , Feuchtmayr, H. , Atkinson, D. , … De Meester, L. (2015). Rapid evolution of thermal tolerance in the water flea *Daphnia* . Nature Climate Change, 5(7), 665.

[ece34550-bib-0026] Gill, J. K. , Arora, J. S. , Kumar, B. S. , Mukhopadhyay, C. S. , Kaur, S. , & Kashyap, N. (2017). Cellular thermotolerance is independent of HSF 1 expression in zebu and crossbred non‐lactating cattle. International Journal of Biometeorology, 61(9), 1687–1693. 10.1007/s00484-017-1350-0 28451769

[ece34550-bib-0027] Godfray, H. C. J. , Beddington, J. R. , Crute, I. R. , Haddad, L. , Lawrence, D. , Muir, J. F. , … Toulmin, C. (2010). Food security: The challenge of feeding 9 billion people. Science, 327(5967), 812–818. 20110467 10.1126/science.1185383

[ece34550-bib-0028] Guindon, S. , & Gascuel, O. (2003). A simple, fast, and accurate algorithm to estimate large phylogenies by maximum likelihood. Systematic Biology, 52(5), 696–704. 10.1080/10635150390235520 14530136

[ece34550-bib-0029] Guinness World Records Limited (2016). Guinness world records. London: Guinness World Records Limited.

[ece34550-bib-0030] Gutierrez‐Alonso, O. , Hawkins, N. J. , Cools, H. J. , Shaw, M. W. , & Fraaije, B. A. (2017). Dose‐dependent selection drives lineage replacement during the experimental evolution of SDHI fungicide resistance in *Zymoseptoria tritici* . Evolutionary Applications., 10, 1055–1066. 10.1111/eva.12511. 29151860 PMC5680630

[ece34550-bib-0031] Habary, A. , Johansen, J. L. , Nay, T. J. , Steffensen, J. F. , & Rummer, J. L. (2017). Adapt, move or die–how will tropical coral reef fishes cope with ocean warming? Global Change Biology, 23(2), 566–577. 10.1111/gcb.13488 27593976

[ece34550-bib-0032] Harbauer, A. B. , Zahedi, R. P. , Sickmann, A. , Pfanner, N. , & Meisinger, C. (2014). The protein import machinery of mitochondria—a regulatory hub in metabolism, stress, and disease. Cell Metabolism, 19(3), 357–372. 10.1016/j.cmet.2014.01.010 24561263

[ece34550-bib-0033] Hessen, D. O. , Daufresne, M. , & Leinaas, H. P. (2013). Temperature‐size relations from the cellular‐genomic perspective. Biological Reviews, 88(2), 476–489. 10.1111/brv.12006 23551915

[ece34550-bib-0034] Hiendleder, S. , Lewalski, H. , & Janke, A. (2008). Complete mitochondrial genomes of *Bos taurus* and *Bos indicus* provide new insights into intra‐species variation, taxonomy and domestication. Cytogenetic and Genome Research, 120(1–2), 150–156. 18467841 10.1159/000118756

[ece34550-bib-0035] Hill, M. E. Jr , Hill, M. G. , & Widga, C. C. (2008). Late Quaternary Bison diminution on the Great Plains of North America: Evaluating the role of human hunting versus climate change. Quaternary Science Reviews, 27(17–18), 1752–1771. 10.1016/j.quascirev.2008.07.002

[ece34550-bib-0036] Hoffmann, A. A. , Sgrò, C. M. , & Kristensen, T. N. (2017). Revisiting adaptive potential, population size, and conservation. Trends in Ecology & Evolution, 32(7), 506–517. 10.1016/j.tree.2017.03.012 28476215

[ece34550-bib-0037] Horsburgh, K. A. , Prost, S. , Gosling, A. , Stanton, J. A. , Rand, C. , & Matisoo‐Smith, E. A. (2013). The genetic diversity of the Nguni breed of African Cattle (*Bos* spp.): Complete mitochondrial genomes of haplogroup T1. PloS One, 8(8), e71956 10.1371/journal.pone.0071956 23977187 PMC3747060

[ece34550-bib-0038] Hunt, M. , Gall, A. , Ong, S. H. , Brener, J. , Ferns, B. , Goulder, P. , … Otto, T. D. (2015). IVA: Accurate de novo assembly of RNA virus genomes. Bioinformatics, 31(14), 2374–2376. 25725497 10.1093/bioinformatics/btv120 PMC4495290

[ece34550-bib-0039] Isbell, F. , Craven, D. , Connolly, J. , Loreau, M. , Schmid, B. , Beierkuhnlein, C. , … Ebeling, A. (2015). Biodiversity increases the resistance of ecosystem productivity to climate extremes. Nature, 526(7574), 574–577. 26466564 10.1038/nature15374

[ece34550-bib-0040] Jia, S. , Zhou, Y. , Lei, C. , Yao, R. , Zhang, Z. , Fang, X. , & Chen, H. (2010). A new insight into cattle's maternal origin in six Asian countries. Journal of Genetics and Genomics, 37(3), 173–180. 10.1016/S1673-8527(09)60035-7 20347826

[ece34550-bib-0041] Johnson, C. N. , Balmford, A. , Brook, B. W. , Buettel, J. C. , Galetti, M. , Guangchun, L. , & Wilmshurst, J. M. (2017). Biodiversity losses and conservation responses in the Anthropocene. Science, 356(6335), 270–275. 28428393 10.1126/science.aam9317

[ece34550-bib-0042] Katoh, K. , Misawa, K. , Kuma, K. I. , & Miyata, T. (2002). MAFFT: A novel method for rapid multiple sequence alignment based on fast Fourier transform. Nucleic Acids Research, 30(14), 3059–3066. 10.1093/nar/gkf436 12136088 PMC135756

[ece34550-bib-0043] Katoh, K. , & Standley, D. M. (2013). MAFFT multiple sequence alignment software version 7: Improvements in performance and usability. Molecular Biology and Evolution, 30(4), 772–780. 10.1093/molbev/mst010 23329690 PMC3603318

[ece34550-bib-0044] Kearse, M. , Moir, R. , Wilson, A. , Stones‐Havas, S. , Cheung, M. , Sturrock, S. , … Thierer, T. (2012). Geneious Basic: An integrated and extendable desktop software platform for the organization and analysis of sequence data. Bioinformatics, 28(12), 1647–1649. 10.1093/bioinformatics/bts199 22543367 PMC3371832

[ece34550-bib-0045] Kim, T. W. , Park, S. , & Sin, E. (2018). At the tipping point: Differential influences of warming and deoxygenation on the survival, emergence, and respiration of cosmopolitan clams. Ecology and Evolution, 8(10), 4860–4866. 10.1002/ece3.4041 29876064 PMC5980464

[ece34550-bib-0046] Klockmann, M. , Gunter, F. , & Fischer, K. (2017). Heat resistance throughout ontogeny: Body size constrains thermal tolerance. Global Change Biology, 23(2), 686–696. 10.1111/gcb.13407 27371939

[ece34550-bib-0047] Lajbner, Z. , Pnini, R. , Camus, M. F. , Miller, J. , & Dowling, D. K. (2018). Experimental evidence that thermal selection shapes mitochondrial genome evolution. Scientific Reports, 8(1), 9500. 29934612 10.1038/s41598-018-27805-3 PMC6015072

[ece34550-bib-0048] Lees, A. M. , Lees, J. C. , Lisle, A. T. , Sullivan, M. L. , & Gaughan, J. B. (2018). Effect of heat stress on rumen temperature of three breeds of cattle. International Journal of Biometeorology, 62(2), 207–215. 10.1007/s00484-017-1442-x 28918576

[ece34550-bib-0049] Lenstra, J. A. , Ajmone‐Marsan, P. , Beja‐Pereira, A. , Bollongino, R. , Bradley, D. G. , Colli, L. , … Ginja, C. (2014). Meta‐analysis of mitochondrial DNA reveals several population bottlenecks during worldwide migrations of cattle. Diversity, 6(1), 178–187. 10.3390/d6010178

[ece34550-bib-0050] Liu, H. , Cai, S. , Liu, J. , & Zhang, H. (2018). Comparative mitochondrial genomic analyses of three chemosynthetic vesicomyid clams from deep‐sea habitats. Ecology and Evolution, 8, 1–12. 10.1002/ece3.4153 PMC6106168 30151147

[ece34550-bib-0051] Loftus, R. T. , MacHugh, D. E. , Bradley, D. G. , Sharp, P. M. , & Cunningham, P. (1994). Evidence for two independent domestications of cattle. Proceedings of the National Academy of Sciences of USA, 91(7), 2757–2761. 10.1073/pnas.91.7.2757 PMC43449 8146187

[ece34550-bib-0052] Machac, A. , Graham, C. H. , & Storch, D. (2018). Ecological controls of mammalian diversification vary with phylogenetic scale. Global Ecology and Biogeography, 27(1), 32–46. 10.1111/geb.12642

[ece34550-bib-0053] Maloney, S. K. , Marsh, M. K. , McLeod, S. R. , & Fuller, A. (2017). Heterothermy is associated with reduced fitness in wild rabbits. Biology Letters, 13(12), 20170521 10.1098/rsbl.2017.0521 29212751 PMC5746534

[ece34550-bib-0054] Marinov, M. , Teofanova, D. , Radoslavov, G. , & Hristov, P. I. (2018). Mitochondrial diversity of Bulgarian native dogs suggests dual phylogenetic origin. PeerJ Preprints, 6, p. e26636v1. 10.7717/peerj.5060 PMC6026455 29967734

[ece34550-bib-0055] Martin, M. (2011). Cutadapt removes adapter sequences from high‐throughput sequencing reads. EMBnet.journal, 17(1), 10 10.14806/ej.17.1.200

[ece34550-bib-0056] Martin, J. M. , Mead, J. I. , & Barboza, P. S. (2018). Bison body size and climate change. Ecology and Evolution, 8(9), 4564–4574. 10.1002/ece3.4019 29760897 PMC5938452

[ece34550-bib-0057] Mazel, F. , Mooers, A. O. , Riva, G. V. D. , & Pennell, M. W. (2017). Conserving phylogenetic diversity can be a poor strategy for conserving functional diversity. Systematic Biology, 66(6), 1019–1027. 10.1093/sysbio/syx054 28595366

[ece34550-bib-0058] McCain, C. M. , & King, S. R. (2014). Body size and activity times mediate mammalian responses to climate change. Global Change Biology, 20(6), 1760–1769. 10.1111/gcb.12499 24449019

[ece34550-bib-0059] McManus, C. , Prescott, E. , Paludo, G. R. , Bianchini, E. , Louvandini, H. , & Mariante, A. S. (2009). Heat tolerance in naturalized Brazilian cattle breeds. Livestock Science, 120(3), 256–264. 10.1016/j.livsci.2008.07.014

[ece34550-bib-0060] Mehla, K. , Magotra, A. , Choudhary, J. , Singh, A. K. , Mohanty, A. K. , Upadhyay, R. C. , … Khan, F. (2014). Genome‐wide analysis of the heat stress response in Zebu (Sahiwal) cattle. Gene, 533(2), 500–507. 10.1016/j.gene.2013.09.051 24080481

[ece34550-bib-0061] Mitchell, D. , Snelling, E. P. , Hetem, R. S. , Maloney, S. K. , Strauss, W. M. , & Fuller, A. (2018). Revisiting concepts of thermal physiology: Predicting responses of mammals to climate change. Journal of Animal Ecology, 87, 956–973. 10.1111/1365-2656.12818 29479693

[ece34550-bib-0062] Nguyen, T. T. , Bowman, P. J. , Haile‐Mariam, M. , Pryce, J. E. , & Hayes, B. J. (2016). Genomic selection for tolerance to heat stress in Australian dairy cattle. Journal of Dairy Science, 99(4), 2849–2862. 10.3168/jds.2015-9685 27037467

[ece34550-bib-0063] Pacifici, M. , Foden, W. B. , Visconti, P. , Watson, J. E. , Butchart, S. H. , Kovacs, K. M. , … Corlett, R. T. (2015). Assessing species vulnerability to climate change. Nature Climate Change, 5(3), 215 10.1038/nclimate2448

[ece34550-bib-0064] Pacifici, M. , Visconti, P. , Butchart, S. H. , Watson, J. E. , Cassola, F. M. , & Rondinini, C. (2017). Species’ traits influenced their response to recent climate change. Nature Climate Change, 7(3), 205 10.1038/nclimate3223

[ece34550-bib-0065] Park, S. D. , Magee, D. A. , McGettigan, P. A. , Teasdale, M. D. , Edwards, C. J. , Lohan, A. J. , … Chamberlain, A. T. (2015). Genome sequencing of the extinct Eurasian wild aurochs, *Bos primigenius*, illuminates the phylogeography and evolution of cattle. Genome Biology, 16(1), 234 10.1186/s13059-015-0790-2 26498365 PMC4620651

[ece34550-bib-0066] Pereira, A. M. F. , Titto, E. L. , Infante, P. , Titto, C. G. , Geraldo, A. M. , Alves, A. , … Almeida, J. A. (2014). Evaporative heat loss in *Bos taurus*: Do different cattle breeds cope with heat stress in the same way? Journal of Thermal Biology, 45, 87–95. 10.1016/j.jtherbio.2014.08.004 25436956

[ece34550-bib-0067] Portner, H. O. , & Knust, R. (2007). Climate change affects marine fishes through the oxygen limitation of thermal tolerance. Science, 315(5808), 95–97. 17204649 10.1126/science.1135471

[ece34550-bib-0068] Posada, D. (2008). jModelTest: Phylogenetic model averaging. Molecular Biology and Evolution, 25(7), 1253–1256. 10.1093/molbev/msn083 18397919

[ece34550-bib-0069] R Core Team (2018). R: A language and environment for statistical computing. Vienna, Austria: R Foundation for Statistical Computing https://www.R-project.org/

[ece34550-bib-0070] Rabouille, C. , & Alberti, S. (2017). Cell adaptation upon stress: The emerging role of membrane‐less compartments. Current Opinion in Cell Biology, 47, 34–42. 10.1016/j.ceb.2017.02.006 28342303

[ece34550-bib-0071] Ramesha, K. P. , Divya, P. , Rao, A. , Basavaraju, M. , Jeyakumar, S. , Das, D. N. , & Kataktalware, M. A. (2016). Assessment of genetic diversity among Malnad Gidda, Punganur and Vechur‐dwarf cattle breeds of India using microsatellite arkers. Indian Journal of Animal Sciences, 86(2), 186–191.

[ece34550-bib-0072] Ronen, R. , Boucher, C. , Chitsaz, H. , & Pevzner, P. (2012). SEQuel: Improving the accuracy of genome assemblies. Bioinformatics, 28(12), i188–i196. 10.1093/bioinformatics/bts219 22689760 PMC3371851

[ece34550-bib-0073] Rozzi, R. (2017). A new extinct dwarfed buffalo from Sulawesi and the evolution of the subgenus *Anoa*: An interdisciplinary perspective. Quaternary Science Reviews, 157, 188–205. 10.1016/j.quascirev.2016.12.011

[ece34550-bib-0074] Sambrook, J. , Fritsch, E. F. , & Maniatis, T. (1989). Molecular cloning: A laboratory manual. New York, NY: Cold Spring Harbour Laboratory Press.

[ece34550-bib-0075] Savolainen, O. , Lascoux, M. , & Merila, J. (2013). Ecological genomics of local adaptation. Nature Reviews Genetics, 14(11), 807 10.1038/nrg3522 24136507

[ece34550-bib-0076] Seebacher, F. , White, C. R. , & Franklin, C. E. (2015). Physiological plasticity increases resilience of ectothermic animals to climate change. Nature Climate Change, 5(1), 61 10.1038/nclimate2457

[ece34550-bib-0077] Slater, G. J. (2015). Iterative adaptive radiations of fossil canids show no evidence for diversity‐dependent trait evolution. Proceedings of the National Academy of Sciences, 112(16), 4897–4902. 10.1073/pnas.1403666111 PMC4413353 25901311

[ece34550-bib-0078] Stothard, P. , & Wishart, D. S. (2004). Circular genome visualization and exploration using CGView. Bioinformatics, 21(4), 537–539. 10.1093/bioinformatics/bti054 15479716

[ece34550-bib-0079] Taye, M. , Lee, W. , Jeon, S. , Yoon, J. , Dessie, T. , Hanotte, O. , … Lee, H. K. (2017). Exploring evidence of positive selection signatures in cattle breeds selected for different traits. Mammalian Genome, 28(11–12), 528–541. 10.1007/s00335-017-9715-6 28905131

[ece34550-bib-0080] Tilman, D. , Clark, M. , Williams, D. R. , Kimmel, K. , Polasky, S. , & Packer, C. (2017). Future threats to biodiversity and pathways to their prevention. Nature, 546(7656), 73 10.1038/nature22900 28569796

[ece34550-bib-0081] Troy, C. S. , MacHugh, D. E. , Bailey, J. F. , Magee, D. A. , Loftus, R. T. , Cunningham, P. , … Bradley, D. G. (2001). Genetic evidence for Near‐Eastern origins of European cattle. Nature, 410(6832), 1088. 11323670 10.1038/35074088

[ece34550-bib-0082] Tucker, C. M. , Davies, T. J. , Cadotte, M. W. , & Pearse, W. D. (2018). On the relationship between phylogenetic diversity and trait diversity. Ecology, 99, 1–7. 10.1002/ecy.2349 29782644

[ece34550-bib-0083] Upadhyay, M. R. , Chen, W. , Lenstra, J. A. , Goderie, C. R. J. , MacHugh, D. E. , Park, S. D. E. , … Van Arendonk, J. A. M. (2017). Genetic origin, admixture and population history of aurochs (*Bos primigenius*) and primitive European cattle. Heredity, 118(2), 169 10.1038/hdy.2017.59 27677498 PMC5234481

[ece34550-bib-0084] Withers, P. C. , Cooper, C. E. , Maloney, S. K. , Bozinovic, F. , & Neto, A. P. C. (2016). Ecological and environmental physiology of mammals, Vol. 5. Oxford: Oxford University Press.

