# Supplementary material for: Size does matter: Parallel evolution of adaptive thermal tolerance and body size facilitates adaptation to climate change in domestic cattle
Source: Ecol Evol. 2018 Oct 5;8(21):10608–20. doi: 10.1002/ece3.4550 (PMC6238145; doi:10.1002/ece3.4550)
Supplement: Supplementary file 1 [file ECE3-8-10608-s001.docx]

**SUPPLEMENTARY INFORMATION**

**Size does matter: Parallel evolution of adaptive thermal tolerance and body size facilitates adaptation to climate change in domestic cattle**

Running head: Parallel evolution of tolerance and dwarfing

Muhammed Elayadeth-Meethal^1, 2, 4*^, Aravindakshan Thazhathu Veettil^1^, Shane K. Maloney^2^, Nichola Hawkins^3^, Tom H. Misselbrook^4^, Sejian Veerasamy ^1, 5^, Jordana Rivero^4^ and Michael R.F. Lee^4, 6^

^1^Kerala Veterinary and Animal Sciences University, Pookode, Wayanad, Kerala 673576, India.

^2^School of Human Sciences, University of Western Australia, Stirling Highway, Crawley 6009, Australia.

^3^Rothamsted Research, Harpenden, Hertfordshire, AL5 2JQ, UK.

^4^Rothamsted Research, North Wyke, Devon EX20 2SB, UK.

^5^ICAR-National Institute of Animal Nutrition and Physiology, Audugodi, Bangalore 560030, India.

^6^University of Bristol, Bristol Veterinary School, Langford, Somerset BS40 5DU, UK.

*Corresponding author: [muhammed@kvasu.ac.in](mailto:muhammed.elayadeth-meethal@rothamsted.ac.uk) (Tel: +91 4936 256919)

**Description:**

I. Materials and Methods

Data analysis- R code

II. Supplementary Figures (1-14)

III. Supplementary tables (1-5)

IV Supplementary references

Data analysis- R code

**Correlation plot and principal component analysis**

The packages used were ggplot2, devtools and ggbiplot.

pairs.panels (DATA[, -1], gap=0, bg= c("red", "green", "blue")[DATA$ Animal], pch=21)

pct<- prcomp(DATA[, -1], center= TRUE, scale= TRUE)

biplot <- ggbiplot (pct, obs.scale = 1, var.scale = 1, groups = DATA$Animal, ellipse = TRUE, circle = TRUE, eclipse.prob=0.95)

biplot <- g + scale_color_discrete(name = '').

biplot <- g + theme (legend.direction = 'horizontal', legend.position = 'top')

**Model definition and analysis of variance**

Repeated measure analysis was done using mixed effect model.The packages used include psych, nlme, car, multcompView, lsmeans, ggplot2 and rcompanion.

The autocorrelation structure was described with the correlation statement.  In this case, corAR1 is used to indicate a temporal autocorrelation structure of order one, often abbreviated as AR (1).  This statement takes the form:
correlation = Structure (form = ~ THI | Animal)

where:

Structure is the autocorrelation structure.

THI is temperature humidity index over successive time

Autocorrelation structure was chosen by following two method

model.a = lme(response variable~ Breed+ Temperture.humidity.index.THI. + Breed*Temperture.humidity.index.THI.,

random = ~1|Animal)

ACF(model.a)

model = lme (Response variable~ Breed + Temperture.humidity.index.THI. + Breed*Temperture.humidity.index.THI., random = ~1|Animal,

correlation = corAR1(form = ~ Temperture.humidity.index.THI.| Animal,

value = ACF model a), method="REML")

Anova (model)

Test of random effects in the model

The random effects in the model was tested by comparing to a model fitted with just the fixed effects and excluding the random effect.

model.fixed = gls(response variable~ Breed + Temperture.humidity.index.THI. + Breed*Temperture.humidity.index.THI.,

method="REML")

anova(model,

model.fixed)

model.null = lme(response variable~ 1,

random = ~1|Animal,

data = TR)

p-value, pseudo R-squared for model, post-hoc analysis, interaction plot and residuals

The nagelkerke function can be used to calculate a p-value and pseudo R-squared value for the model

Nagelkerke (model,

model.null)

leastsquare = lsmeans (model,

pairwise ~ Breed: Temperture.humidity.index.THI.,

adjust="tukey”)

cld(leastsquare,

alpha= 0.05,

Letters = letters,

adjust = "tukey")

Sum = groupwiseMean (response variable~ Breed+ Temperture.humidity.index.THI.,

conf = 0.95,

digits = 3,

traditional = FALSE,

percentile= TRUE)

Sum

pd = position_dodge(.2)

ggplot (Sum, aes(x =Temperture.humidity.index.THI.,

y =Mean,

color = Breed)) +

geom_errorbar(aes(ymin=Percentile.lower,

ymax=Percentile.upper),

width=.2, size=0.7, position=pd) +

geom_point(shape=15, size=4, position=pd) +

theme_bw() +

theme (axis.title = element_text(face = "bold")) +

ylab ("response variable)")

x = residuals (model)

plotNormalHistogram(x)

plot (fitted (model),

residuals(model))

**Supplementary Figures (1-14)**


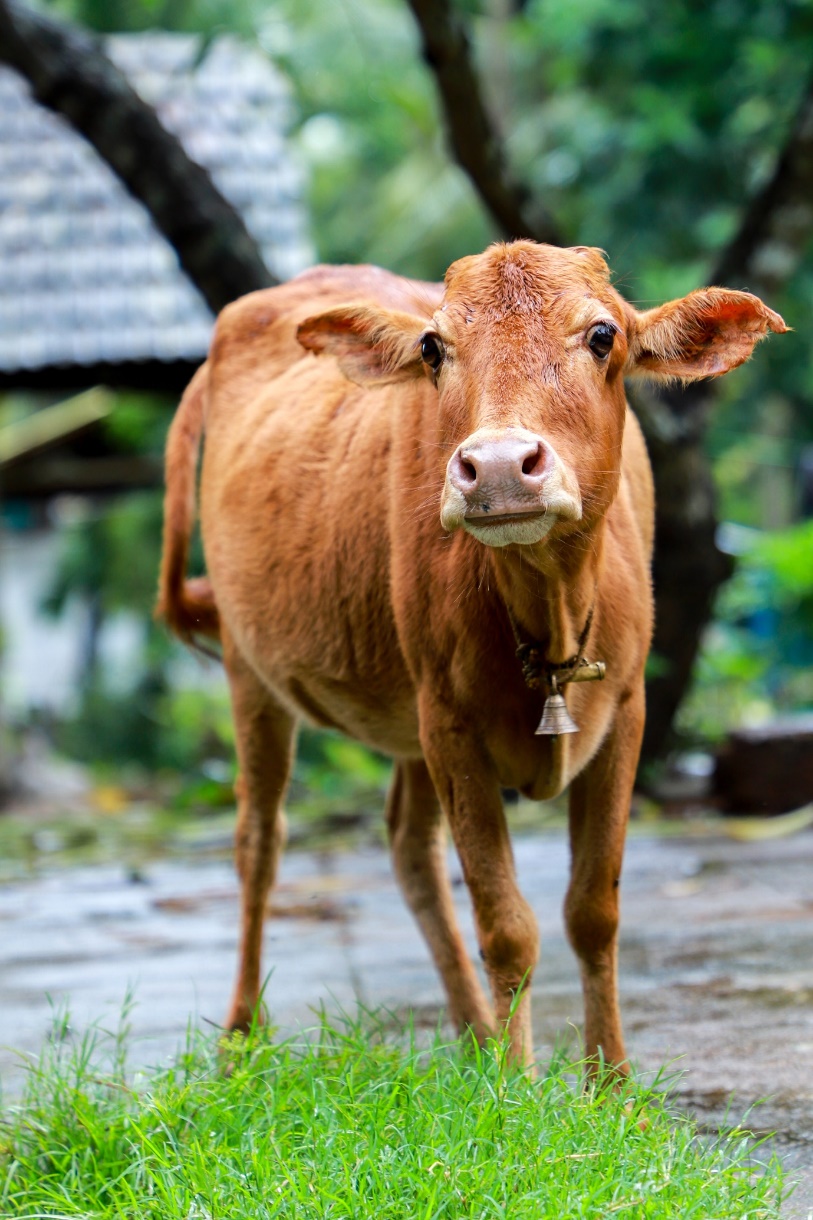


.

**Supplementary figure 1. Manikyam-** the **s**mallest Vechur cattle (Guinness World Records, 2016).


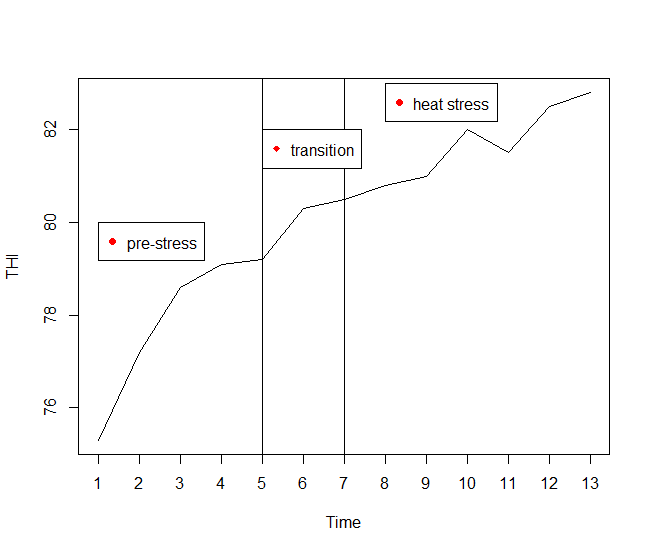


**Supplementary figure 2**.Temperature Humidity Index (THI) during the study period at half-hour intervals beginning at 8.00 am (1=8.00 am, 2=8.30 am….13=2.00 pm). Pre-stress (8.00 am to 10 am- THI= 75.3 to 79.2, transition (10 am to 11 am-THI=79.2 to 80.5, heat stress-11 am to 2 pm-THI= 80.5 to 82.8).


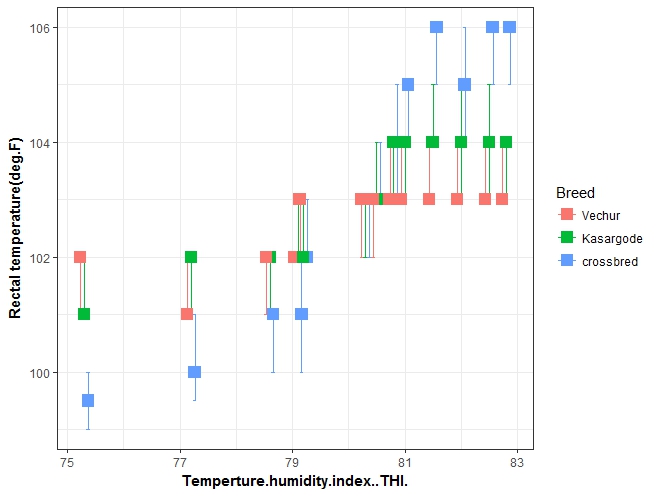


**Supplementary figure 3.** Mean rectal temperature of Vechur, Kasargode and crossbred cattle plotted as a function of temperature humidity index with successive progression of stresswith residuals plotted.

.
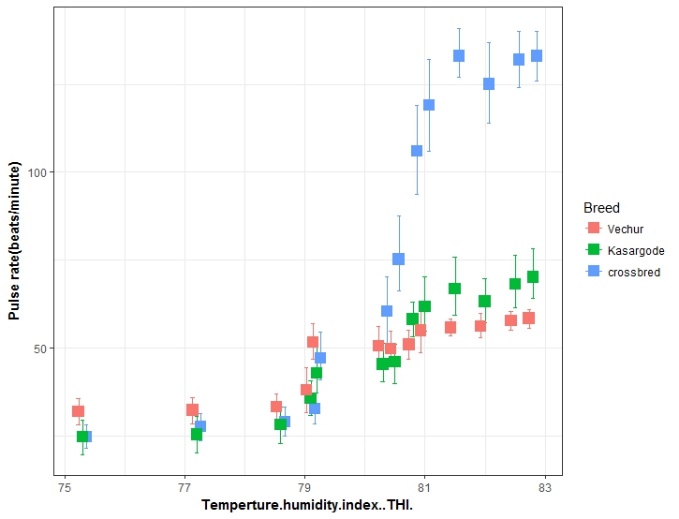


**Supplementary figure 4.** Mean pulse rate of Vechur, Kasargode and crossbred cattle plotted as a function of temperature humidity index with successive progression of stress


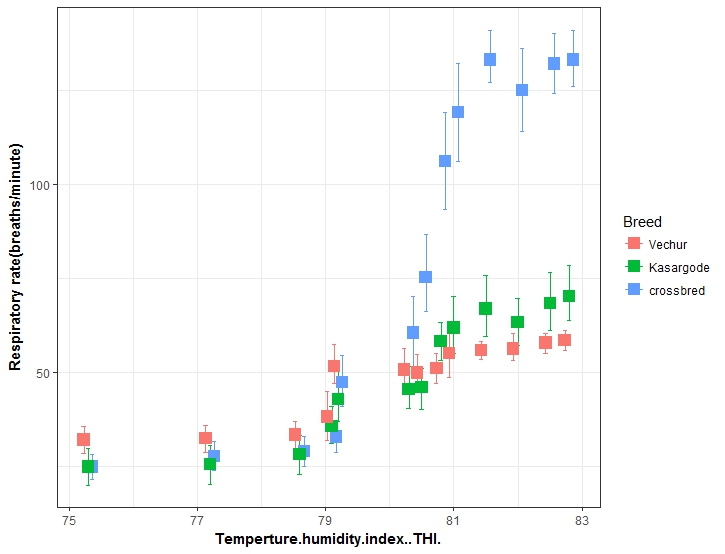


**Supplementary figure 5.** Mean respiratory rate of Vechur, Kasargode and crossbred cattle plotted as a function of temperature humidity index with successive progression of stress. Note that crossbred animals were unable to return to normalcy while Vechur and Kasargode animals did.


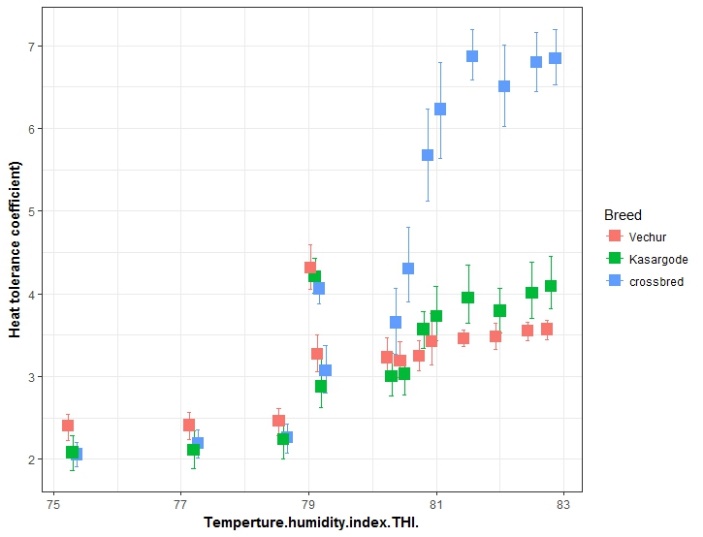


**Supplementary figure 6.** Mean heat tolerance coefficient (HTC) of Vechur, Kasargode and crossbred cattle plotted as a function of temperature humidity index with successive progression of stress. Note the significantly low HTC in Vechur, Kasargode and high HTC in crossbred during heat stress.


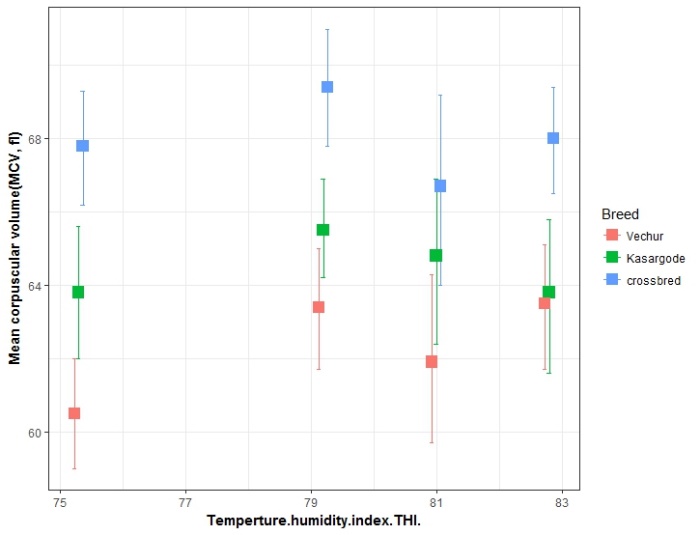


**Supplementary figure 7.**Mean Corpuscular Volume (MCV) of Vechur, Kasargode and crossbred cattle plotted as a function of temperature humidity index with successive progression of stress. Note low MCV of Vechur and Kasargode and higher MCV in crossbred indicating small red blood cell size in dwarf cattle, which we propose as a mechanism of dwarfing.

**
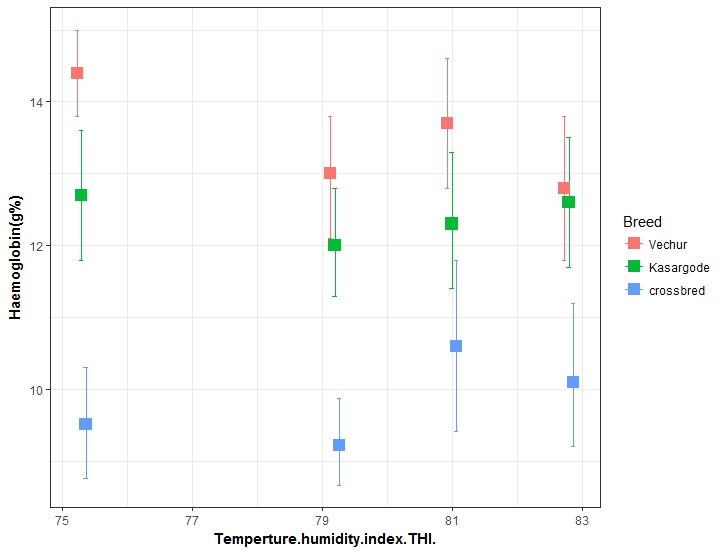
**

**Supplementary figure 8.** Mean Haemoglobin (HB) of Vechur, Kasargode and crossbred cattle plotted as a function of temperature humidity index with the 4 sample times during a day are shown. Note the significantly low HB in crossbred and high HB in Vechur, Kasargode during heat stress.


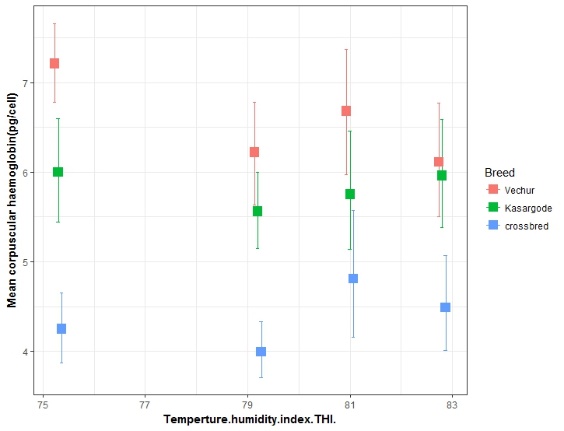


**Supplementary figure 9.** Mean Corpuscular haemoglobin (MCH)of Vechur, Kasargode and crossbred cattle plotted as a function of temperature humidity index with successive progression of stress. Note the significantly low MCH in Vechur, Kasargode and high MCH in crossbred during heat stress.


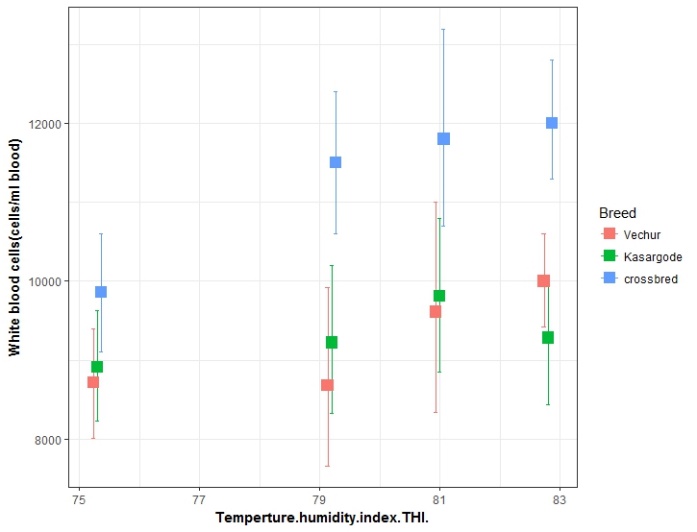


**Supplementary figure 10.** Mean White blood cell count (WBC) count of Vechur, Kasargode and crossbred cattle plotted as a function of temperature humidity index with successive progression of stress.


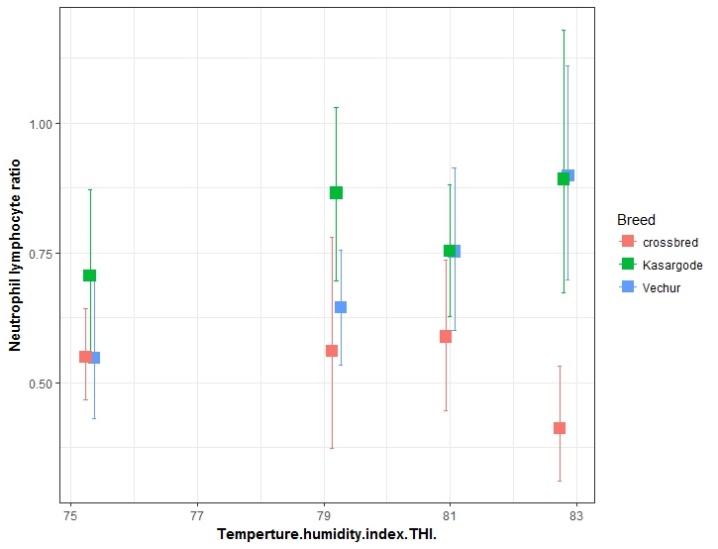


**Supplementary figure 11.** Mean neutrophil-lymphocyte (N/L ratio) of Vechur, Kasargode and crossbred cattle plotted as a function of temperature humidity index with successive progression of stress. Note similar N/L ratio of Vechur, Kasargode and crossbred cattle during EH and significant deviation to a low level in crossbred during heat stress.

**
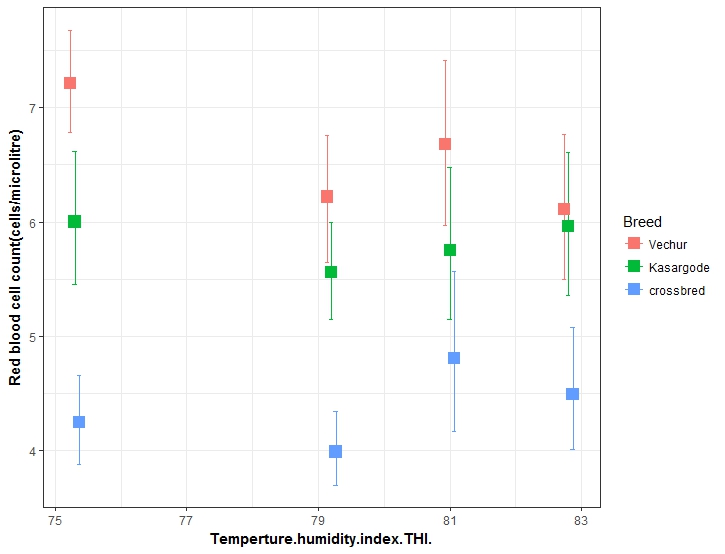
**

**Supplementary figure 12.** Red Blood Cell (RBC) count of Vechur, Kasargode and crossbred cattle plotted as a function of temperature humidity index with successive progression of stress.


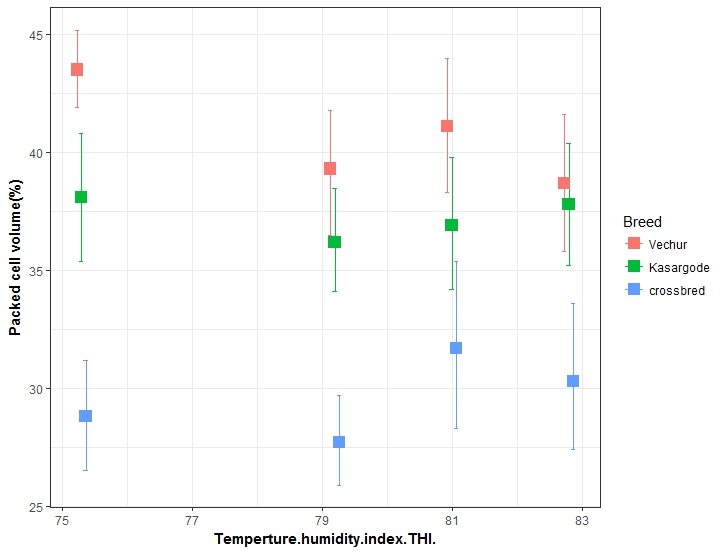


**Supplementary figure 13.** Mean packed cell volume (PCV) Vechur, Kasargode and crossbred cattle plotted as a function of temperature humidity index with successive progression of stress.


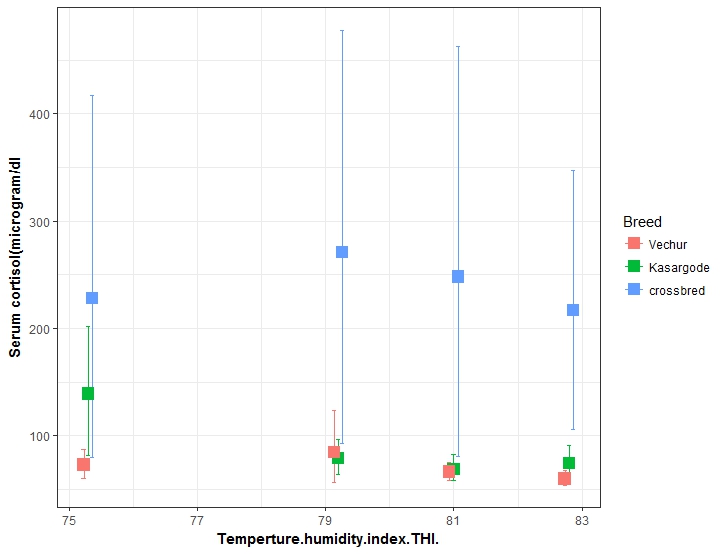


**Supplementary figure 14.** Mean serum cortisol of Vechur, Kasargode and crossbred cattle plotted as a function of temperature humidity index with successive progression of stress. Note the significantly high serum cortisol in crossbred during heat stress.

**Supplementary tables (1-5)**

**Supplementary table 1.** Target genes and primer sequences used in QRT-PCR

| **Name** | **Sequence (5’→ 3’)** | **Ta**  **(^°^ C)** | **Expected size (bp)** | **Source sequence** |
| --- | --- | --- | --- | --- |
| HSP70 | F-AAGAAGAAGGTGCTGGACAAGTGC  R-TCCTCTTGTGCTCAAACTCGTCCT | 60 | 91 | NM203322 |
| ATP1A1 | F-TCCTCATCGGCATCATTGTAGCCA  R-AGCCTCCAGGTTCTTCACTAAGCA | 60 | 122 | NM1076798 |
| ACTB | F-GCAAGCATAAAGTGCTGTGGGTGT  R-GCACATGCAGAAGAGTGCAAGGAA | 60 | 181 | NM173979 |
| GAPDH | F-TGGAGAAACCTGCCAAGTATG  R-TGAGTGTCGCTGTTGAAGTC | 60 | 127 | NM1034034 |

**Supplementary table 2.** Results of the principal component analysis showing the effects on vital physiological parameters in response to heat stress.

|  | PC1 | PC2 | PC3 | PC4 |
| --- | --- | --- | --- | --- |
| Rectal temperature | 0.5186 | -0.2880 | 0.5027 | 0.6289 |
| Respiratory rate | 0.5266 | 0.1944 | 0.4428 | -0.6991 |
| Pulse rate | 0.4731 | 0.7146 | -0.4313 | 0.2818 |
| Temperature humidity index | 0.4795 | -0.6071 | -0.6043 | -0.1904 |
| Standard deviation | 1.73 | 0.70 | 0.58 | 0.41 |
| Proportion of variance | 0.75 | 0.12 | 0.08 | 0.04 |
| Cumulative proportion | 0.75 | 0.87 | 0.95 | 1.00 |

**Supplementary table 3.** Results of the linear mixed effect models for physiological, haematological, serum cortisol and relative expression of candidate genes in Vechur, Kasargode and crossbred cattle. Significant p- values are given in bold

|  | F | Df | Df_res_ | p | Adjusted-R^2^ |
| --- | --- | --- | --- | --- | --- |
| Rectal temperature | 2.318 | 2 | 387 | 0.0999 | 0.0067 |
| Respiratory rate | 50.97 | 2 | 387 | **<0.001** | 0.2044 |
| Pulse rate | 26.42 | 2 | 387 | **<0.001** | 0.1156 |
| Total haemoglobin  concentration | 58.57 | 2 | 117 | **<0.001** | 0.498 |
| Mean corpuscular volume | 31.57 | 2 | 117 | **<0.001** | 0.3394 |
| Serum cortisol | 12.39 | 2 | 117 | **<0.001** | 0.1607 |
| Relative expression-  -HSP 70 | 4.064 | 2 | 33 | **0.0264** | 0.149 |
| -ATP1A1 | 0.8388 | 2 | 33 | 0.4412 | -0.0093 |
| -GAPDH | 0.06284 | 2 | 24 | 0.9392 | -0.0777 |
| -ACTB | 3.611 | 2 | 24 | **0.0425** | 0.1673 |

**Supplementary table 4.** Linear mixed effect models for vital physiological parameters (rectal temperature, respiratory rate and pulse rate) for Vechur, Kasargode and crossbred cattle. Respiratory rate values were log transformed. Significant p- values are given in bold

|  | Estimate | SE | t-value | p | F _(Df, Dfres)_ | Adjusted-R^2^ |
| --- | --- | --- | --- | --- | --- | --- |
| **Temperature+ Respiratory rate** | | | | | | |
| Intercept | 33.10 | 0.23 | 143.71 | **<0.001** | 271.2  (3, 386) | 0.6757 |
| Respiratory rate | 1.53 | 0.05 | 28.27 | **<0.001** |  |  |
| Kasargode | 0.53 | 0.07 | 7.72 | **<0.001** |  |  |
| Vechur | 0.29 | 0.07 | 4.36 | **<0.001** |  |  |
| **Temperature+ Pulse rate** | | | | | | |
| Intercept | 37.14 | 0.154 | 240.45 | **<0.001** | 94.35 (3,386) | 0.4186 |
| Pulse rate | 0.02 | 0.001 | 16.58 | **<0.001** |  |  |
| Kasargode | 0.45 | 0.093 | 4.80 | **<0.001** |  |  |
| Vechur | -0.18 | 0.089 | -1.98 | **0.0475** |  |  |

**Supplementary table 5.** Origin and sub-haplogroup affiliation of mitogenomes considered in this study.

| Name | Species | Haplo group | GenBank ID | Reference |
| --- | --- | --- | --- | --- |
| Goat | *Capra hircus* |  | AF533441 | (Hiendleder *et al.*, 2008) |
| Sheep | *Ovis aries* |  | AF010406 | (Hiendleder *et al.*, 2008) |
| Buffalo | *Bubalis bubalis* |  | NC006295 | (Hiendleder *et al.*, 2008) |
| Yak | *Bos grunniens* |  | NC 006380 | (Hiendleder *et al.*, 2008) |
| Dwrf cattle (Srilanka) | *Bos indicus* | I2 | AF492350 | (Hiendleder *et al.*, 2008) |
| Vechur | *Bos indicus* | I1 | MF667929 | This study |
| Wayanad | *Bos indicus* | I1 | MF667931 | This study |
| Kasargode | *Bos indicus* | I2 | MF667930 | This study |
| CB (crossbred) | *B. indicus* × *B. taurus* |  | MF667932 | This study |
| Cattle | *Bos indicus* | I1 | NC005971 | (Hiendleder *et al.*, 2008) |
| Nellore | *Bos indicus* | I1 | AY126697 | (Hiendleder *et al.*, 2008) |
| Cattle | *Bos taurus* | R | FJ971087 | (Achilli *et al.*, 2009) |
| Cattle | *Bos taurus* | R | FJ971086 | (Achilli *et al.*, 2009) |
| Cattle | *Bos taurus* | R | FJ971085 | (Achilli *et al.*, 2009) |
| Cattle | *Bos taurus* | R | FJ971084 | (Achilli *et al.*, 2009) |
| Korean cattle | *Bos taurus* |  | DQ124389 | (Hiendleder *et al.*, 2008) |
| Auroch | *Bos primigeneous* |  | NC013996 | (Hiendleder *et al.*, 2008) |
| Auroch | *Bos primigeneous* |  | GU985279 | (Hiendleder *et al.*, 2008) |
| Cattle | *Bos taurus* | Q | FJ971082 | (Achilli *et al.*, 2009) |
| Cattle | *Bos taurus* | Q | FJ971083 | (Achilli *et al.*, 2009) |
| Cattle | *Bos taurus* | Q | FJ971081 | (Achilli *et al.*, 2009) |
| Cattle | *Bos taurus* | Q | FJ971080 | (Achilli *et al.*, 2009) |
| Cattle | *Bos taurus* | T1 | KF163061 | (Achilli *et al.*, 2009) |
| Cattle | *Bos taurus* | T3 | V00654 | (Achilli *et al.*, 2009) |
| White Park cattle | *Bos taurus* |  | KC153972 | (Hiendleder *et al.*, 2008) |
| Cattle | *Bos taurus* |  | AF492351 | (Hiendleder *et al.*, 2008) |
| Northern European cattle | *Bos taurus* |  | AY676856 | (Hiendleder *et al.*, 2008) |
| Cabannia cattle | *Bos taurus* | T2 | EU177851 | (Achilli *et al.*, 2008) |
| Cattle | *Bos indicus* | I2 | EU177870 | (Achilli *et al.*, 2008) |
| Cattle | *Bos indicus* | I2 | EU177869 | (Achilli *et al.*, 2008) |
| Cattle | *Bos indicus* | I1 | EU177868 | (Achilli *et al.*, 2008) |

**REFERENCES**

Achilli A, Bonfiglio S, Olivieri A *et al.* (2009) The multifaceted origin of taurine cattle reflected by the mitochondrial genome. PloS one*,***4**, e5753.

Achilli A, Olivieri A, Pellecchia M *et al.* (2008) Mitochondrial genomes of extinct aurochs survive in domestic cattle. Current Biology*,***18**, R157-R158.

Guinness World Records Limited , 2016. *Guinness world records*, London, England.

Hiendleder S, Lewalski H, Janke A (2008) Complete mitochondrial genomes of *Bos taurus* and *Bos indicus* provide new insights into intra-species variation, taxonomy and domestication. Cytogenetic and Genome Research*,***120**, 150-156.
